# Experimental and theoretical investigations into the stability of cyclic aminals

**DOI:** 10.3762/bjoc.12.221

**Published:** 2016-10-31

**Authors:** Edgar Sawatzky, Antonios Drakopoulos, Martin Rölz, Christoph Sotriffer, Bernd Engels, Michael Decker

**Affiliations:** 1 Pharmazeutische und Medizinische Chemie, Institut für Pharmazie und Lebensmittelchemie, Julius-Maximilians-Universität Würzburg, Am Hubland, D-97074 Würzburg, Germany; 2 Institut für Physikalische und Theoretische Chemie, Julius-Maximilians-Universität Würzburg, Emil-Fischer-Straße 42, D-97074 Würzburg, Germany

**Keywords:** hydrolysis, kinetics, molecular mechanics, natural products, quantum mechanics

## Abstract

**Background:** Cyclic aminals are core features of natural products, drug molecules and important synthetic intermediates. Despite their relevance, systematic investigations into their stability towards hydrolysis depending on the pH value are lacking.

**Results:** A set of cyclic aminals was synthesized and their stability quantified by kinetic measurements. Steric and electronic effects were investigated by choosing appropriate groups. Both molecular mechanics (MM) and density functional theory (DFT) based studies were applied to support and explain the results obtained. Rapid decomposition is observed in acidic aqueous media for all cyclic aminals which occurs as a reversible reaction. Electronic effects do not seem relevant with regard to stability, but the magnitude of the conformational energy of the ring system and p*K*_a_ values of the N-3 nitrogen atom.

**Conclusion:** Cyclic aminals are stable compounds when not exposed to acidic media and their stability is mainly dependent on the conformational energy of the ring system. Therefore, for the preparation and work-up of these valuable synthetic intermediates and natural products, appropriate conditions have to be chosen and for application as drug molecules their sensitivity towards hydrolysis has to be taken into account.

## Introduction

**Figure 1:**
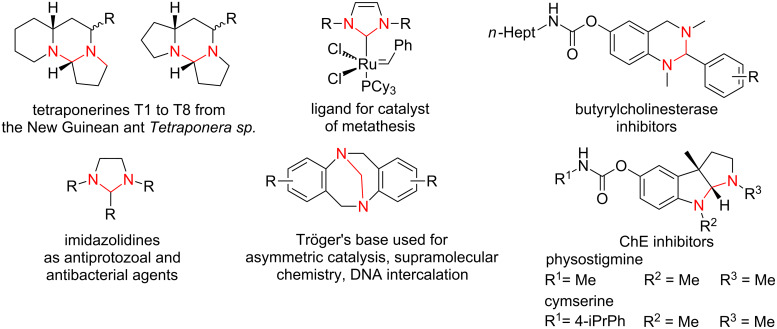
Compounds described in the literature containing an aminal core for various applications. The aminal structure is highlighted in red.

The aminal system (*N*,*N*-acetal) is the structurally equivalent analogue of the *O*,*O*-acetal. In the literature this moiety is found as a core element in various important structures, for example in the naturally occurring alkaloids tetraponerine T1 to T8 from the venom of the New Guinean ant *Tetraponera sp.* [1–2]. It is also present in ligands of ruthenium-based catalysts for metathesis [3], in imidazolidines acting as antiprotozoal and antibacterial agents [4–5], in Tröger’s base derivatives with diverse applications [6–14] (e.g., asymmetric catalysis, supramolecular chemistry, DNA intercalation, etc.) or in synthetic tetrahydroquinazolines as cholinesterase (ChE) inhibitors [15–17] as well as ChE inhibitors based on the scaffold of the naturally occurring alkaloid physostigmine from the calabar bean *physostigma venenosum* [18–19] (Figure 1). This is just a small overview and selection of examples out of numerous compounds incorporating the aminal system as the essential structural feature.

**Scheme 1:**
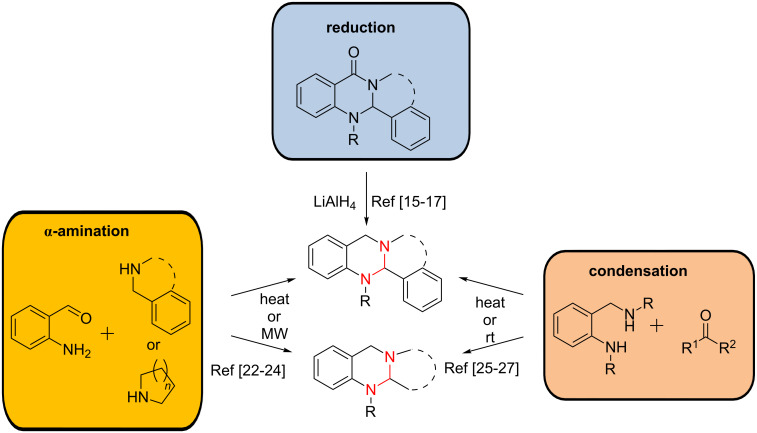
Synthetic approaches for the formation of the tetrahydroquinazoline moiety. Dashed lines indicate both cyclized or non-cyclized compounds and the aminal structure is highlighted in red.

Especially the aminal-bearing tetrahydroquinazoline system is of great interest as a structure derived from the quinazolinone and quinazoline cores. These represent privileged structures with various applications in medicinal chemistry [20–21]. The syntheses of tetrahydroquinazolines are well described using different approaches: the majority relies on the direct α-amination of *o*-aminobenzaldehydes with heating or microwave irradiation [22–24], by condensation of diamines with aldehydes or ketones yielding bicyclic structures [25–27], or by the reduction of the corresponding dihydroquinazolinones [15–17] (Scheme 1).

**Scheme 2:**
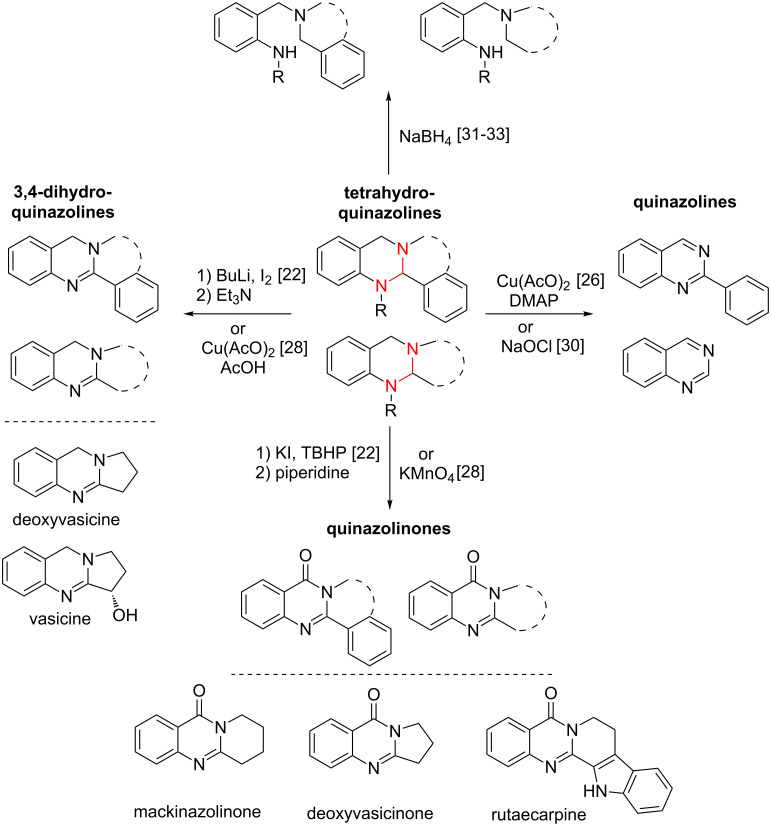
Oxidation and reduction reactions of tetrahydroquinazolines. Dashed lines indicate both cyclized or non-cyclized compounds and the aminal structure is highlighted in red.

The aminal templates obtained can be used as starting materials for the synthesis of a broad spectrum of diverse structures. Oxidation reactions with KMnO_4_ or a mixture of potassium iodide and *tert*-butyl hydroperoxide (TBHP) give access to quinazolinones and have been reported for the synthesis of the naturally occurring alkaloids deoxyvasicinone, mackinazolinone or rutaecarpine [22,28] (Scheme 2). Besides total oxidation of the aminal core, also a partial oxidation towards 3,4-dihydroquinazolines is possible for the case that the oxidation is promoted either by Cu(AcO)_2_ or by a mixture of elemental iodine and BuLi. These methods allow the synthesis of the partially unsaturated alkaloids vasicine or deoxyvasicine in good yields [22,28] (Scheme 2). Furthermore, copper-catalysed reactions or oxidation with sodium hypochlorite were also described to yield the aromatic quinazoline core [26,29–30] (Scheme 2). Besides all the oxidation reactions described, also reductive conditions applying NaBH_4_ onto the tetrahydroquinazoline-based aminal systems were investigated, thereby providing the possibility to “open” the rigid aminal core gaining access to sterically more flexible compounds [31–33] (Scheme 2).

Although the chemistry of aminals – especially concerning tetrahydroquinazolines – is in the focus of current research and includes their preparation and modification, it is remarkable that there is only little focus on the pH stability of these compounds in aqueous media. In general, the aminal moiety is known to undergo (similar to the corresponding *O*,*O*-acetals) acidic hydrolysis and can be considered as stable only within a certain pH range. This is of enormous importance especially for aminal-functionalities-bearing compounds which might be exposed to an acidic environment, e.g., drugs that are orally applied and get into direct contact with gastric acid. The pH stability of the aminal system is also a key property for synthetic approaches of such compounds to prevent undesired decomposition during reaction or work-up. Knowledge about the pH stability can therefore help to improve the yield during a reaction, or to prevent complete degradation of the product by applying inappropriate conditions. To our knowledge, there is only little data [34–35] describing the stability of the aminal moiety and no study conducted a systematic investigation. The conditions for stability of aminals – especially in tetrahydroquinazolines – are highly important because of the increasing relevance of such compounds in medicinal chemistry.

**Figure 2:**
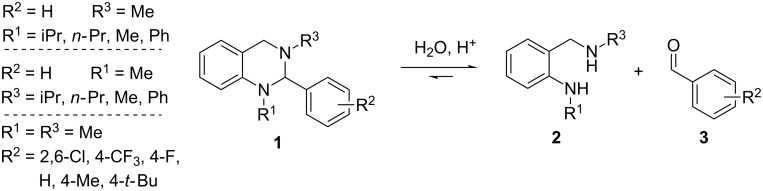
Hydrolysis of the aminal core of tetrahydroquinazolines **1** into the corresponding diamines **2** and aldehydes **3**.

In the present study, we synthesized and modified tetrahydroquinazolines of the general structure **1** to investigate the pH stability of the aminal core in dependence of the steric and electronic properties of different groups at the N-1 and N-3 nitrogen atoms, as well as at the aromatic residue in position 2 (Figure 2). For that purpose, two residues were always kept constant while altering the third one. To investigate the effect of decreasing electron density at the nitrogen sites onto the stability of the aminal core, iPr, *n-*Pr, Me, and Ph moieties, respectively, were chosen as substituents for both the N-1 and N-3 nitrogen atoms. At the same time the iPr and Ph moieties served as bulkier substituents for investigations into steric effects. Similar to the N-1 and N-3 nitrogen atoms, 4-*t-*Bu, 4-Me, 4-F and 4-CF_3_ groups were incorporated into the aromatic site at position 2 to investigate the influence of decreasing electron density at this position. These substituents were chosen as their influence onto the aromatic residue is mainly determined by inductive effects and therefore no pronounced mesomeric effect has to be considered. In general, to estimate the electron-donating effect of all substituents (and therefore the change in electron density at all sides), we used the data reported by Craig [36] for the aromatic site and the data reported by Topliss [37] for the side chains, respectively. A disubstituted 2,6-dichloro compound was also synthesized to study the influence of steric interactions at this site of the structure, as 2,6-disubstitution of the phenyl ring prevents coplanar orientation.

All synthesized compounds were exposed to aqueous media at defined pH values and the time-dependent hydrolysis into the corresponding diamines **2** and aldehydes **3** (Figure 2) was quantified by reversed-phase HPLC. Based on these data, quantum mechanical calculations revealed that the hydrolysis of the test compounds is a thermodynamically driven process. Interestingly, we could show that this equilibrium is strongly dependent on the applied reaction conditions and that a change from an acidic to a neutral environment can well induce the formation of tetrahydroquinazolines, instead of their hydrolysis. We also were able to determine differences in the hydrolysis rate caused by the respective substituents and found the decrease in stability of these compounds to be a result of enthalpic effects.

## Results and Discussion

### Synthesis of test compounds

**Scheme 3:**
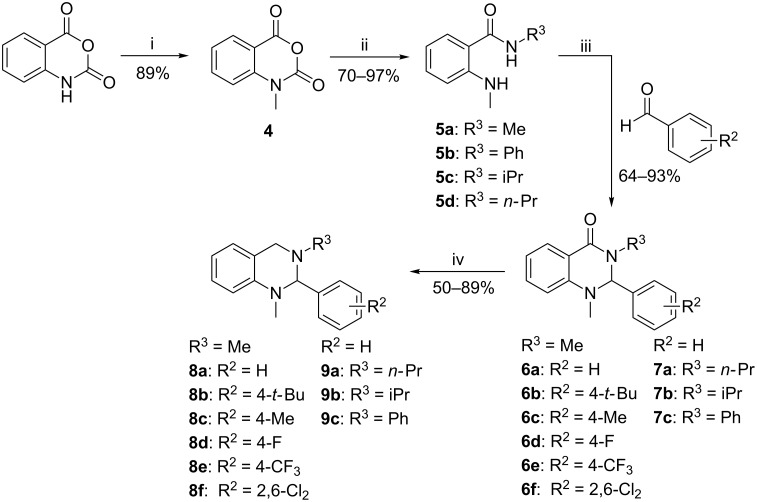
Reagents and conditions: (i) MeI, DIPEA, DMAc, 40 °C, 24 h; (ii) R^1^-NH_2_ or MeNH_3_Cl and Et_3_N, DMF, 40–120 °C, 3–6 h; (iii) AcOH, 70 °C, 1–4 h; (iv) LiAlH_4_, THF, 70 °C, 1–3 h.

The synthesis of tetrahydroquinazolines substituted at the phenyl ring as well as at the 3-*N* nitrogen atom was achieved in 4 steps (Scheme 3). Briefly, isatoic anhydride was methylated using MeI to yield compound **4**, followed by formation of amides **5a–d** using the corresponding free amines or their salts. Cyclization towards dihydroquinazolinones **6a–f** and **7a–c** was performed using benzaldehyde derivatives under acidic conditions with moderate to excellent yields. The tetrahydroquinazoline target compounds **8a–f** and **9a–c** were finally obtained by reduction with LiAlH_4_.

**Scheme 4:**
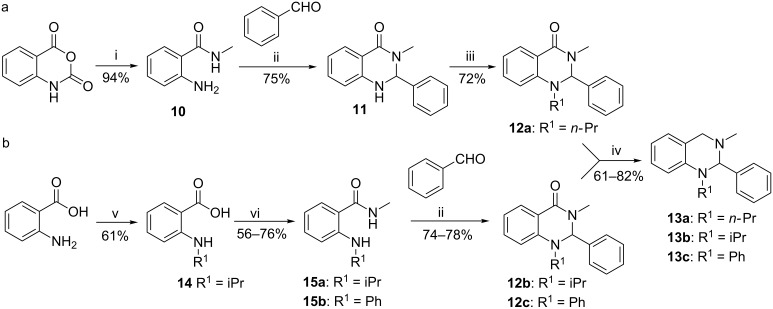
Reagents and conditions: (a) (i) MeNH_3_Cl, Et_3_N, DMF, 70 °C, 3 h; (ii) AcOH, 70 °C, 4 h; (iii) *n-*PrBr, *t-*BuOK, DMF, 110 °C, 16 h; (iv) LiAlH_4_, THF, 70 °C, 2–3 h; (b) (v) 1) acetone, MeOH, 80 °C, 5 h; 2) NaBH_4_, rt, 3 h; (vi) MeNH_3_Cl, Et_3_N, EDCI, HOBt, DMF, 70 °C, 8–14 h.

The introduction of different substitution patterns at the N-1 nitrogen atom was achieved using two pathways as shown in Scheme 4. Because the direct alkylation of isatoic anhydride in the first reaction step with different alkyl halides failed (Scheme 3), amide **10** was synthesized using isatoic anhydride and methylammonium hydrochloride (Scheme 4a) followed by cyclization under acidic conditions with benzaldehyde to yield dihydroquinazolinone **11**. Unfortunately, the introduction of substituents at the N-1 in **11** with alkyl halides was only successful with *n-*PrBr in the presence of the strong non-nucleophilic base *t-*BuOK to give compound **12a**. The target compound **13a** could then be obtained by reduction of **12** with LiAlH_4_.

As the substitution reactions using **11** failed with iPrI, a second synthetic pathway was pursued to alter the substituents at the N-1 nitrogen atom of the tetrahydroquinazoline core (Scheme 4b). Thus, anthranilic acid was alkylated by reductive amination with acetone and NaBH_4_ in two steps to yield the isopropyl-substituted derivative **14**. The derivative **14** and the commercially available *N*-phenylanthranilic acid were converted to amides **15a**,**b** under standard conditions. Final cyclization with benzaldehyde yielded dihydroquinazolinones **12b**,**c** which after reduction with LiAlH_4_ afforded the desired tetrahydroquinazolines **13b**,**c** (Scheme 4).

### Stability experiments

**Figure 3:**
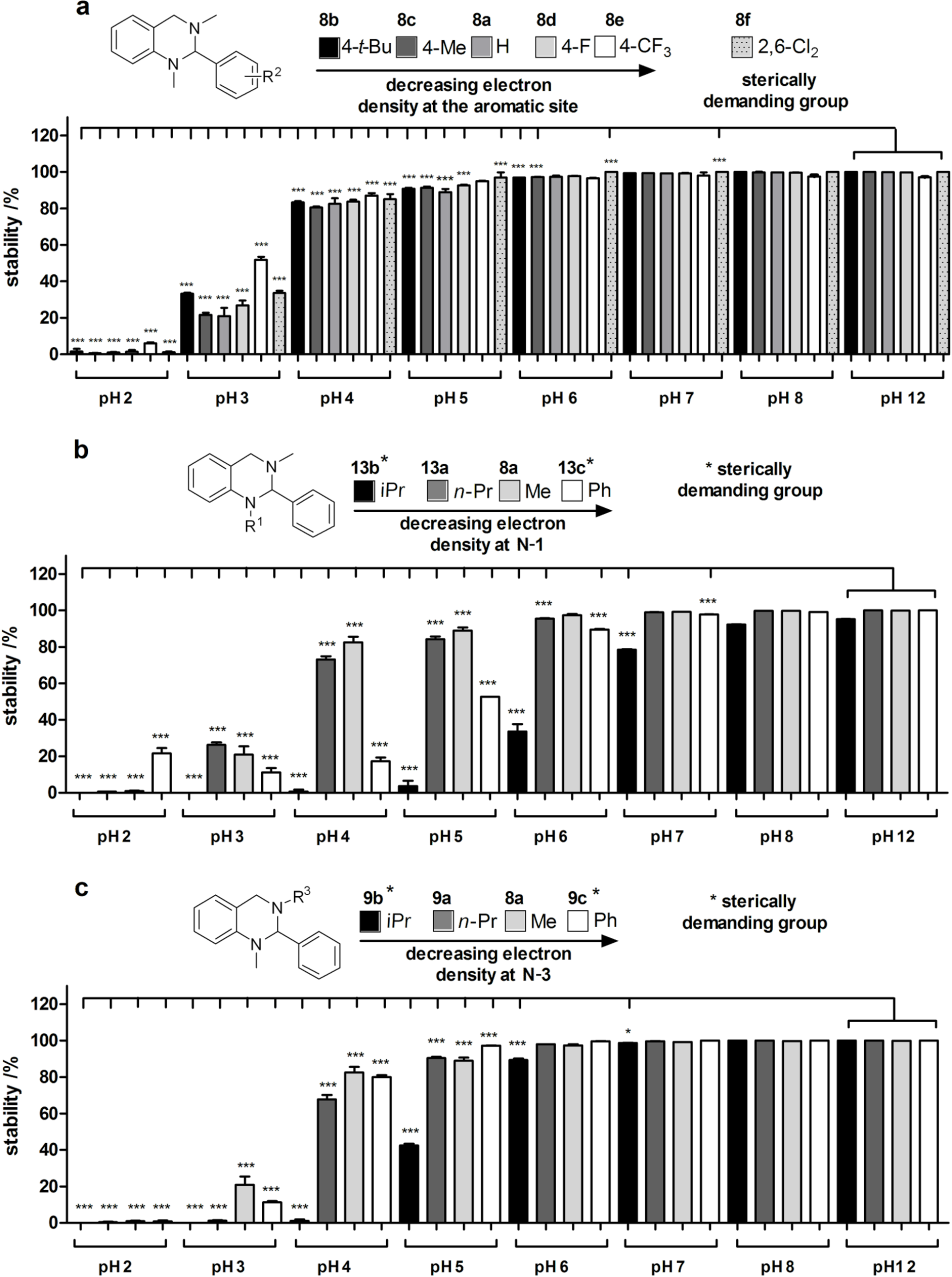
pH-Stability test of the aminal core toward hydrolysis in dependency of different substitution pattern at (**a**) the 2-phenyl residue, (**b**) the N-1 position and (**c**) the N-3 position. Experiments were performed in triplicate (mean + SD).

All compounds were exposed to phosphate buffered aqueous systems with defined pH values between pH 2 and pH 12 for 1 h to investigate the hydrolysis of the aminal core. After the treatment, reversed-phase HPLC analyses were performed to determine the ratio of intact tetrahydroquinazoline **1** and the corresponding aldehyde **3** as cleavage product using calibration curves (for a detailed description see Supporting Information File 1). The results of this study are summarized in Figure 3a–c.

Compounds **8a–f** (Figure 3a) showed no differences in the extent of hydrolysis depending on their substitution pattern. However, a general trend for all of the test compounds was observed: Compounds **8a**–**f** are stable in a basic or neutral environment down to pH 6 and slowly decomposed at pH 4–5 with less than 20% of hydrolysis after 1 h. Interestingly, at pH 3 the aminal system is significantly hydrolysed by more than 50%, and at pH 2 decomposition of the test compounds is rapidly taking place resulting in complete degradation of the aminal system. These results clearly show a significant pH dependency for hydrolysis of the aminal core which is accelerated in increasingly acidic media. Different substitution patterns at N-1 (Figure 3b) significantly alter the sensitivity of the aminal core towards hydrolysis. While a methyl group (compound **8a**) and an *n-*Pr residue (compound **13a**) did not alter the hydrolysis rate compared to all compounds of the series **8** (Figure 3a), the iPr residue (**13b**) as well as the Ph residue (**13c**) increased hydrolysis of the test compounds: The least stable compound **13b** was found to be completely hydrolysed already at pH 5 and also at pH 6 more than 50% of **13b** decomposed. In contrast, compound **13c** (Ph moiety) completely hydrolysed at pH 4 and showed ~50% hydrolysis at pH 5. Different substituents at the *N*-3 position (Figure 3c) showed no pronounced effects on the pH-dependent decomposition of *n*-Pr (**9a**), Me (**8a**) and a Ph (**9c**) group, respectively. Only the introduction of an iPr residue (**9b**) at *N*-3 led to an increased hydrolysis rate with complete degradation of **9b** already at pH 4. Finally, the 1,2-dihydroquinazolinone compound **6a** was also tested for its stability towards hydrolysis (data not shown). This compound did not undergo any decomposition in the tested pH range after 1 h.

The stability analyses shown in Figure 3 have to be regarded as snapshots after 1 h of incubation time only. Therefore, the kinetics for this reaction were investigated in greater detail. On the assumption that hydrolysis of tetrahydroquinazolines **1** into the corresponding diamines **2** and aldehydes **3** is a reversible reaction (cf. Figure 2), the velocity *v* of the reaction can be described in dependency of time *t* as follows:

[1]
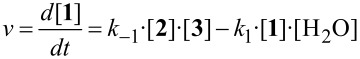


Furthermore, 1) if the conversion of **2** and **3** into **1** is suppressed when [H_2_O] >> [**2**] and [**3**], the term k_−1_·[**2**]·[**3**] can be neglected and 2) if water is used as solvent and therefore remains approximately constant during the reaction, the constant *k*_2_ = *k*_1_·[H_2_O] can be introduced. The velocity can then be described as a pseudo-first-order kinetic:

[2]
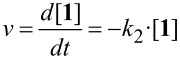


Finally, rearrangement and integration gives an exponential function from which *k*_2_ can be calculated:

[3]
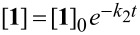


**Figure 4:**
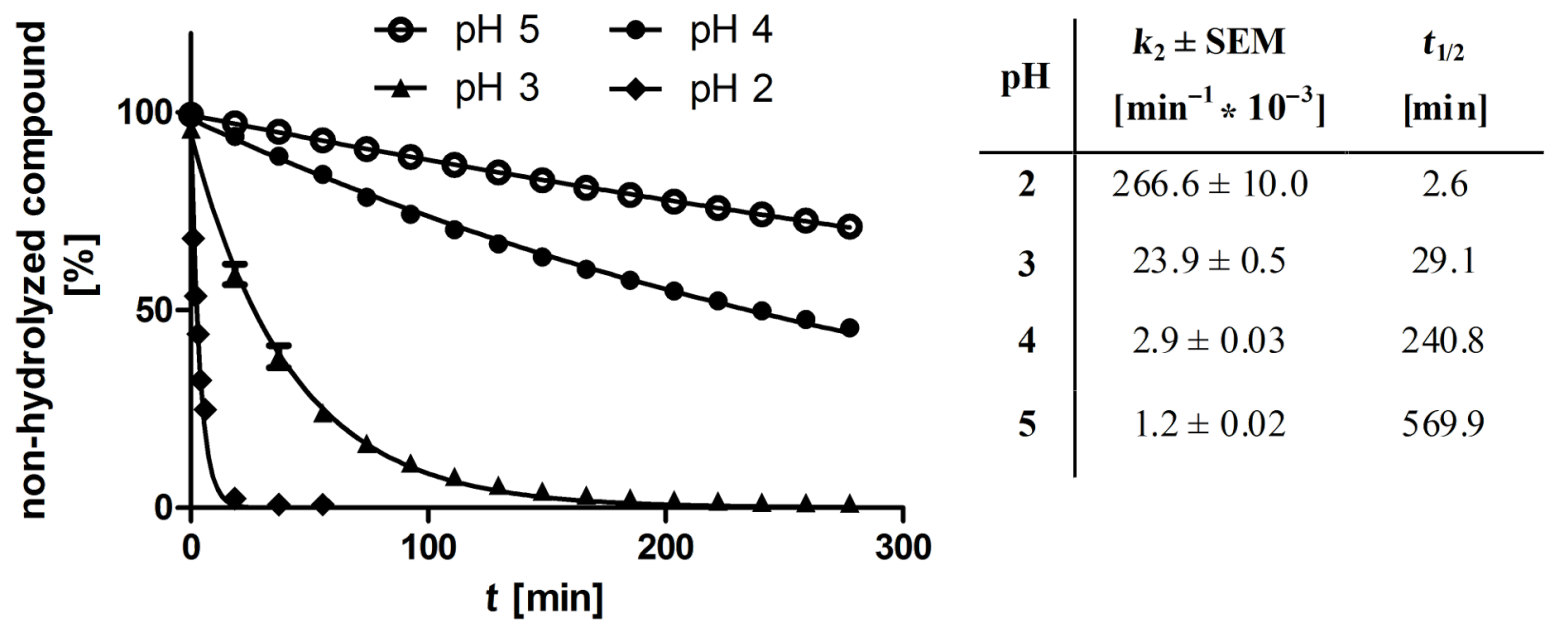
Kinetic analysis of hydrolysis of reference compound **8a** in dependency of different pH values and calculation of kinetic parameters. Curves and *k*_2_ values were calculated assuming a pseudo-first-order kinetic as described in Equation 3. All experiments were performed in triplicate (mean ± SEM).

Kinetic analysis of reference compound **8a** revealed exponential first order kinetic (Figure 4) from which *k*_2_ was calculated.

Interestingly, the comparison of the kinetic parameters showed a ~10-fold increase of the *k*_2_ value from pH 4 to pH 3 and also from pH 3 to pH 2 and therefore a 10-fold increase in velocity of the hydrolysis per pH unit. Only between pH 5 and pH 4 an increase by a factor of 2 is observed. Furthermore, it is remarkable that the half-life time of compound **8a** at pH 2 is only 2.6 min and therefore complete hydrolysis of this compound is expected to occur within a couple of minutes.

### Computational studies and discussion

With regard to the results obtained from the pH-dependent stability test, two factors for hydrolysis need to be investigated. First, the influence of an acidic or a basic environment for the hydrolysis has to be determined due to different hydrolysis rates at different pH values. Second, the influence of different substitution patterns of the test compounds should be verified regarding the altered sensitivity towards hydrolysis for some compounds (**9b** and **13b**,**c**).

Initially, we determined the protonation pattern of the test compounds, as protonation of the tetrahydroquinazoline system is expected to be an essential factor of hydrolysis induction. Theoretically, protonation might occur at the anilinic N-1, the aliphatic N-3 or by double protonation of N-1 and N-3. Therefore the p*K*_a_ values of both nitrogens of the test compounds were predicted using the empirical algorithm of MoKa [38] and validated with density functional theory (DFT) energy calculations of all protonation patterns for compounds **8a** and **13b** (cf. Supporting Information File 1). We used the B3LYP-D3 functional [39–41] in combination with the cc-PVTZ basis sets [42–43] and all computations were performed with TURBOMOLE [44]. Solvent effects were mimicked by the Screening Model (COSMO) for water [45]. As expected, N-3 was found to be more basic for all test compounds, with the exception of **9c**. Therefore, in the following text, only the molecular forms with protonation of the aliphatic nitrogen N-3 will be discussed.

**Figure 5:**
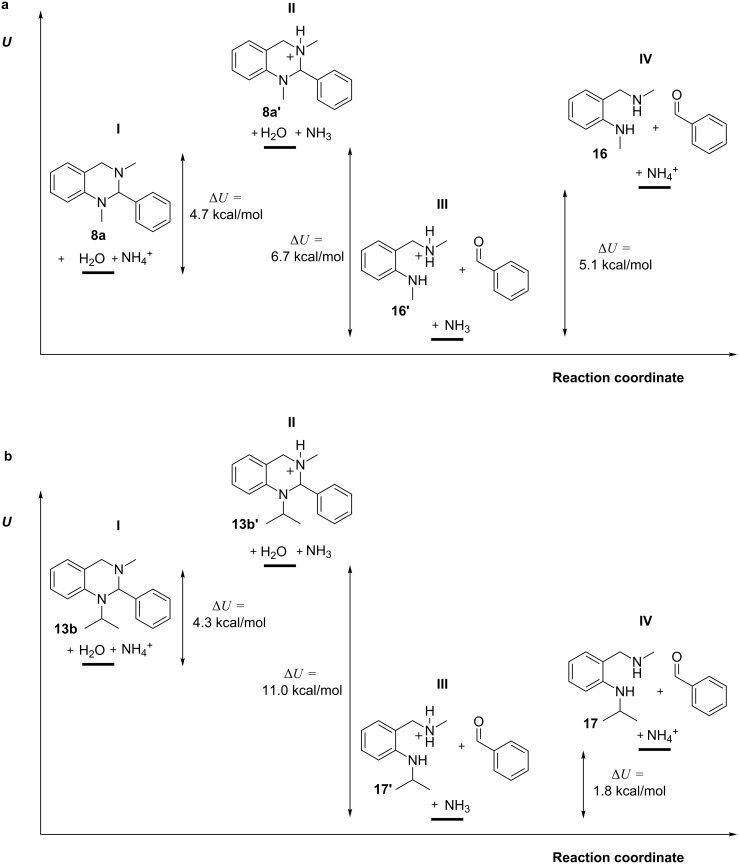
Differences in energy along the reaction coordinate using the functional B3LYP-D3 for the hydrolysis of (a) compound **8a** and (b) compound **13b** in aqueous environment.

To explore the influence of a basic or an acidic reaction environment on hydrolysis, QM single point energy calculations for the hydrolysis of compound **8a** and the least stable compound **13b** were performed. The calculations included the neutral (**8a** and **13b**) and the single protonated species **8a’** and **13b’** (-’- indicates protonation at N-3 throughout) of all reactants and products (cf. Supporting Information File 1). The computations include NH_4_^+^ as proton donor and one water molecule was taken into account for the stoichiometry of the reaction. Interestingly, plotting the data in water (Figure 5) as well as in the gas phase (cf. Supporting Information File 1) for all relevant fragments revealed an exothermic reaction (−6.7 kcal/mol in water) for the hydrolysis of **8a’** (**II** in Figure 5a) into the protonated fragments (**III** in Figure 5a), while hydrolysis of the neutral form **8a** (**I** in Figure 5a) undergoes an endothermic reaction (3.1 kcal/mol in water) into the neutral fragments (**IV** in Figure 5a). This indicates that the fragments show greater stability when protonated, while interestingly, the aminal structure is more stable when it is non-protonated. Therefore, protonation of this compound due to an acidic environment might well shift the equilibrium from the non-hydrolysed compound in neutral media, to its fragments in acidic media (Figure 5a). This implies that formation of tetrahydroquinazolines from their fragments is thermodynamically favoured in a neutral or basic environment. In fact, this is in agreement with data from literature reporting the synthesis of tetrahydroquinazolines to take place in organic solvent [25–27] as well as in non-acidic aqueous media [46]. Indeed, we tried the condensation of the respective diamine **16** and benzaldehyde towards compound **8a** in water and in acetonitrile as solvent. In both cases, complete condensation was observed toward compound **8a**. In addition, as larger amounts of benzaldehyde and diamine **16** in water are not soluble, also a solvent mixture of water/acetonitrile 1:1 was used to dissolve all reactants and to exclude solubility effects on the condensation reaction in water (for details cf. Supporting Information File 1). Interestingly, for the least stable compound **13b** (Figure 5b), the exothermicity of the reaction of the protonated tetrahydroquinazoline (**II** in Figure 5b) into its fragments (**III** in Figure 5b) increased to −11 kcal/mol in aqueous medium. This might explain the increased hydrolysis rate of this compound in acidic media compared to compound **8a’**. In contrast to **8a**, the hydrolysis of the neutral tetrahydroquinazoline **13b** (**I** in Figure 5b) into its neutral fragments (**IV** in Figure 5b) is also exothermic (−4.9 kcal/mol in water), which indicates the hydrolysis of this compound to take place in acidic as well as in neutral environment. These observations are in agreement with the described stability experiments of compound **13b**, which was found to degrade also in neutral environment at pH 7 (cf. Figure 3b). It should also be taken into account that calculations of charged compounds in solution are less accurate than the corresponding calculations of neutral ones. Takano and Houk showed that the mean absolute deviation for single point energy calculations of charged molecules in a continuum solvent model is 3–5 kcal/mol, while 1–3 kcal/mol were computed for neutral molecules [47]. Due to the fact that hydrolysis of tetrahydroquinazolines is an equilibrium reaction, the energy differences between the different states are within the range of 10 kcal/mol, which is close to the accuracy of the method for charged molecules. Therefore, the calculation results should be approached in a manner of showing a trend, rather than expecting exact values.

**Figure 6:**
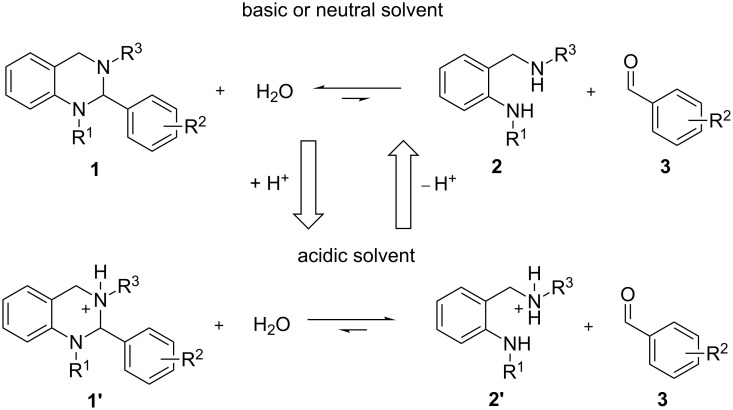
Reaction equilibrium between tetrahydroquinazoline **1**, the corresponding diamine **2** and aldehyde **3** in basic or neutral media as well as for the protonated tetrahydroquinazoline **1’** and the corresponding diamine **2’** and aldehyde **3** in acidic media.

Taking this data together, it is predicted and experimentally proven, that basic reaction conditions shift the equilibrium towards the formation of tetrahydroquinazolines of the general structure **1** from the respective diamines and aldehydes, while acidic conditions promote hydrolysis of the protonated tetrahydroquinazolines **1’** (Figure 6). Therefore, compounds with an increased basicity, like those with a branched alkyl chain at the nitrogen (e.g., compound **9b**), are more sensitive towards hydrolysis due to their accelerated protonation which induces hydrolysis.

**Figure 7:**
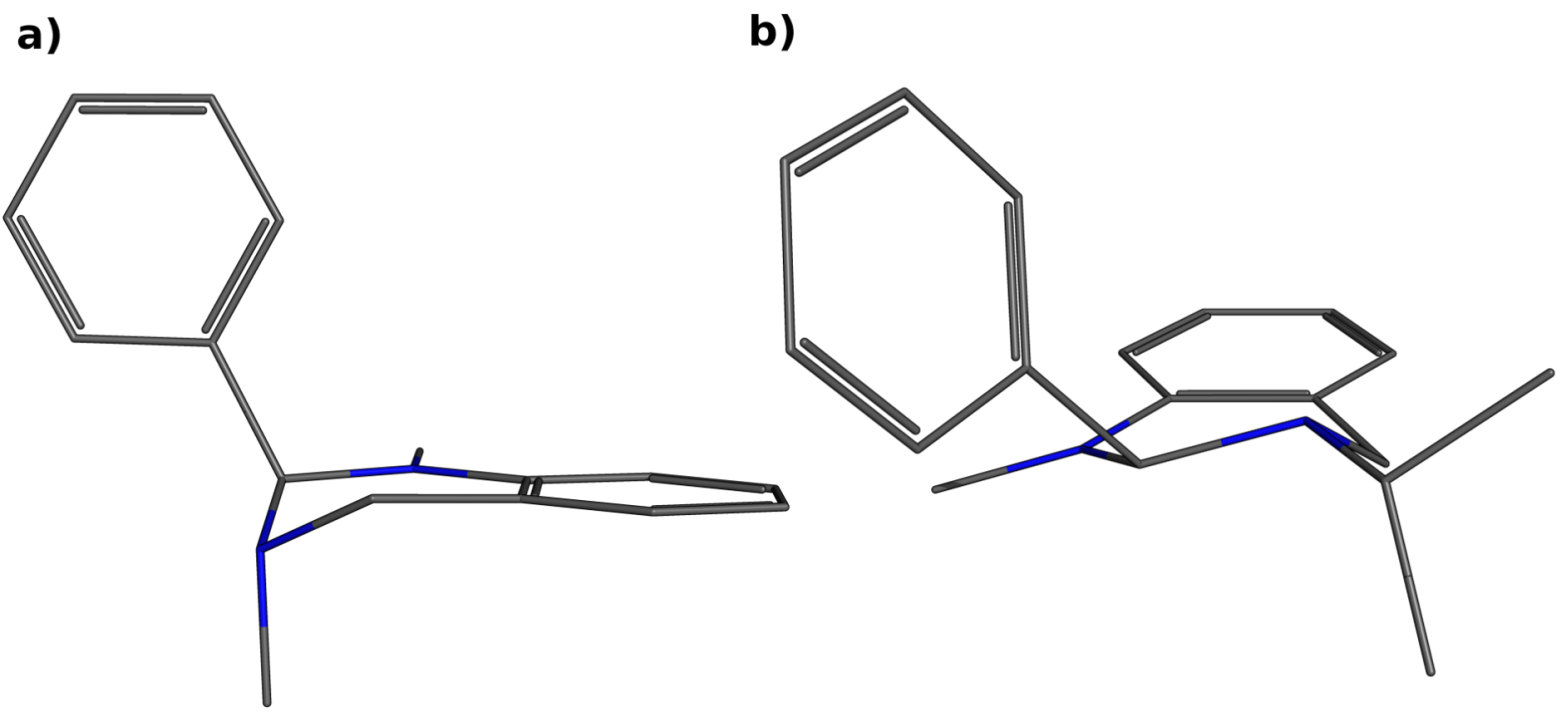
Minimum energy conformers in their neutral form with (a) an axial orientation of the phenyl system exemplarily shown for compound **8a** and (b) an equatorial orientation of the phenyl substituent exemplarily shown for **9b**.

Considering the influence of different substitution patterns for an increased hydrolysis rate of some compounds (**9b** and **13b**,**c**), a systematic conformational search [48] for all compounds was performed to investigate possible differences in the minimum energy conformation of both the neutral and the protonated forms. The conformational search revealed that the majority of all compounds (neutral form: **8a–c**,**e**, **9a**, **13a**–**c**; protonated form: **8a’–e’**, **9a’**, **13a’–c’**), shared the same minimum energy conformer in which the phenyl ring in position 2 and the N-3 side group are in anti-axial orientation (exemplarily shown for compound **8a** in Figure 7a) while the other compounds (neutral form: **8d**,**f**, **9b**,**c**; protonated form: **8f’**, **9b’**,**c’**) adhere to a conformer of minimal energy where the residues are in equatorial position (exemplarily shown for compound **9b** in Figure 7b). Both conformations of minimum energy found in this study are in agreement with crystal structures reported in the literature [46,49–52] (CCDC reference numbers for anti-axial motif: 177049 [46], 717617 [49], 778079 [50], 722943 [51], 820149 [52]; and for equatorial motif: 177050, 177052 [46]).

Interestingly, the least stable compound **13b** and the significantly more stable compound **8e** were found to share the same anti-axial conformer in the neutral and in the N-3 protonated form. Furthermore, the 2,6-dichloro compound **8f** exhibits the same behaviour in terms of stability as the other compounds of the **8** series, even though it adheres to the equatorial minimum energy motif, both in the neutral and in the N-3 protonated form. In conclusion, a comparison of the conformational search and the experimental data of the hydrolysis experiments proves no coherence between the decreased sensitivity on hydrolysis of **9b** and **13b**,**c** due to a different conformation of minimal energy; especially as **13b**,**c** and **13b’**,**c’** are following anti-axial conformation, while **9b** and **9b’** are following equatorial conformation.

To further investigate the increased hydrolysis rates of **9b** and **13b**,**c**, respectively, energetic differences within the aminal system of these compounds were explored. The cyclic tetrahydroquinazoline system is higher in energy by its conformation in comparison to the non-cyclic diamines and bulky substituents, e.g., Ph or iPr residues, might additionally increase the energy in this system (e.g., through strain), thus contributing to its enhanced sensitivity towards hydrolysis. Therefore, a comparison of the energy of the aminal core of the reference compound **8a** with compounds **9b** and **13b**,**c** was performed using Molecular Mechanics (MM). For this purpose, the conformer of minimal energy for compounds **9b** and **13b**,**c** was kept frozen, while the N-side groups were changed into a methyl residue. The resulting structures were therefore identical with compound **8a**, thus differences in potential energy are attributed to the conformational energy of the tetrahydroquinazoline core. This procedure was followed for all the aforementioned compounds in their neutral and the N-3 protonated form. The relative potential energy of the modified compounds (**13b_mod**, **13c_mod** and **9b_mod**) was higher in all cases compared to compound **8a** (Table 1) suggesting a correlation of increased conformational energy of the ring system and increased sensitivity toward hydrolysis. This increase in energy might well be attributed to different geometrical effects including ring strain, strain of the angles or steric repulsion of the side groups. It might be possible that an increased ring strain at the nitrogens of the tetrahydroquinazoline system could increase the basicity of these compounds and therefore accelerate the induction of hydrolysis, although previous studies showed that the relationship between the magnitude of ring strain and the resulting nitrogen basicity is not straightforward [53–54]. Probably more interesting, the hydrolysis of cyclic geminal ethers was recently reported to be drastically accelerated by introduction of sterically demanding side groups through reduction of the activation barrier [55]. These results are consistent with the herein reported data supporting a preferred elimination of the aldehyde fragment from the respective tetrahydroquinazoline in compounds with increased ring energy. Nevertheless, compound **13c_mod** was computed to be the least stable compound although compound **13b** was experimentally proven to be less stable. Therefore, we assume that additional enthalpic or entropic effects might also be involved in altering the stability of the ring system and an increase in conformational energy might be only one part of the puzzle.

**Table 1:** Calculated differences in potential energy (*U*) of the modified compounds compared to compound **8a** in water with MM. Differences were calculated by Δ*U* = *U*(**Cpd_mod** or **Cpd_mod’**) – *U*(**8a** or **8a’**).

	Δ*U* [kcal/mol]				
	**8b_mod**	**8f_mod**	**9b_mod**	**13b_mod**	**13c_mod**

neutral	0.74	1.47	2.09	3.05	5.27

protonated	0.73	1.61	2.46	3.50	5.22

In addition, as a proof of concept, also the energy differences between **8a** and the modified compound **8b_mod** as well as the sterically more demanding compound **8f_mod** were calculated. As expected, in the case of **8b_mod** the difference to **8a** is negligible (Δ*U* < 1 kcal/mol). For the sterically more demanding compound **8f_mod** the energy difference of the protonated form is 1.61 kcal/mol, which is still a moderate value and places it well below compound **9b**. Obviously, an exact correlation of this simple measure with the hydrolytic instability is not to be expected, given the limitations of the molecular mechanical approach and the fact that the hydrolysis rate of the investigated aminal system is not only a function of increased conformational energy but also dependent on other thermodynamic factors, as mentioned above. Nevertheless, a clear trend can be recognized, indicating that the conformational energy of the ring system is at least an important if not the major contribution to the hydrolytic instability of the investigated systems.

## Conclusion

The aminal core is a common structural element in various medicinally relevant compounds and naturally occurring alkaloids. However, this system suffers from hydrolysis and therefore might decompose due to inappropriately applied reaction or work-up conditions in synthesis, or when being exposed to an acidic environment, e.g., by gastric acid when administered orally.

To systematically investigate the pH-dependent hydrolysis of the aminal system, tetrahydroquinazolines **8a–f**, **9a–c** and **13a–c** were synthesised and exposed to buffered aqueous media with defined pH values. A general trend was observed for all compounds with an accelerated hydrolysis rate as a function of decreasing pH value. Additional density functional calculations revealed that protonation of the N-3 nitrogen can induce hydrolysis into the corresponding fragments. Therefore, compounds with a higher p*K*_a_ value at the N-3 nitrogen might increase the hydrolysis sensitivity of these compounds due to their faster protonation, like for compound **9b**. Computational studies as well as experimental data revealed that the formation of tetrahydroquinazolines is favoured when exposing their fragments (diamine and aldehyde) to an environment where no protonation occurs (mostly due to a basic solvent) and therefore the reaction equilibrium can be shifted to one or the other side by protonating or deprotonating the relevant reactants. Furthermore, different substitution patterns in position 2 (series **8**) of the aromatic system did not affect the stability because of changes in electron density (**8a**–**e**) or bulkiness (**8f**) of the substituents.

Interestingly, hydrolysis experiments revealed an accelerated decomposition for compounds **9b** and **13b**,**c**. To investigate these findings, minimum conformational energy calculations were conducted. We found that all compounds adhere to two groups of conformers in accordance with crystal structures reported in literature. Changing the minimum energy conformers of **9b** and **13b**,**c** into the reference structure **8a** revealed an increase in ring energy which might accelerate their hydrolysis. However, additional entropic and enthalpic effects may also influence the stability of such compounds that should be investigated in further studies.

## Supporting Information

File 1 Detailed synthetic procedures, spectral data, stability analyses and computational investigations.
