# Supplementary material for: Experimental and theoretical investigations into the stability of cyclic aminals
Source: Beilstein J Org Chem. 2016 Oct 31;12:2280–92. doi: 10.3762/bjoc.12.221 (PMC5238614; doi:10.3762/bjoc.12.221)
Supplement: File 1 — Detailed synthetic procedures, spectral data, stability analyses and computational investigations. [file Beilstein_J_Org_Chem-12-2280-s001.pdf]

Supporting Information

for

Experimental and theoretical investigations into the stability of cyclic aminals

Edgar Sawatzky[1], Antonios Drakopoulos[1], Martin Rölz[1], Christoph Sotriffer[1], Bernd Engels[2], and Michael Decker[1]*

Address: [1]Pharmazeutische und Medizinische Chemie, Institut für Pharmazie und Lebensmittelchemie, Julius-Maximilians-Universität Würzburg, Am Hubland, D-97074 Würzburg, Germany and [2]Institut für Physikalische und Theoretische Chemie, Julius-Maximilians-Universität Würzburg, Emil-Fischer-Straße 42, D-97074 Würzburg, Germany

Email: Michael Decker - Michael.Decker@uni-wuerzburg.de

*Corresponding author

Detailed synthetic procedures, spectral data, stability analyses and computational investigations

Contents:

Experimental section S3

Chemistry S3

General S3

General reaction procedures S3

Synthesis and experimental data S4

Stability test S17

Preparation of phosphate buffered systems S17

Hydrolysis experiments S17

Aminal condensation experiments S18

Computational section S20

Materials and methods S20

MoKa calculations S21

QM calculations S22

Absolute energy of all reactants S22

Determining the protonation pattern S23

QM summary S24

MM conformational search S25

MM Ring strain comparison in gas phase S28

Additional references S30

Experimental section

Chemistry

General

Common reagents and solvents were obtained from commercial suppliers and used without any further purification. Tetrahydrofuran (THF) was distilled from sodium/benzophenone under argon atmosphere. Reaction progress was monitored using analytical thin layer chromatography (TLC) on precoated silica gel GF254 plates (Macherey-Nagel GmbH & Co. KG, Düren, Germany) and spots were detected under UV light (254 nm) or by staining with iodine. Nuclear magnetic resonance spectra were performed with a Bruker AV-400 NMR instrument (Bruker, Karlsruhe, Germany) in DMSO-d_6 or $CDCl_3$. Chemical shifts are expressed in ppm relative to $CDCl_3$ or DMSO-d_6 (7.26/2.50 and 77.16/39.52 ppm for ^{1}H and ^{13}C NMR, respectively). Melting points were determined in open capillaries on a Büchi B-540 without any further correction. For purity and reaction analyses, analytical HPLC was performed on a system from Shimadzu equipped with a DGU-20A3R controller, LC20AB liquid chromatograph, and a SPD-20A UV/Vis detector. The stationary phase was a Synergi 4U fusion-RP (150 × 4.6 mm) column (Phenomenex, Aschaffenburg, Germany). As mobile phase, water (phase A) and MeOH (phase B) were used with 1 mL/min. (conc. B: 5% → 90% from 0 to 8 min; 90% from 8 to 13 min; 90% → 5% from 13 to 15 min; 5% from 15 to 18 min.) ESI mass spectral data were acquired on a Shimadzu LCMS-2020.

General reaction procedures

General amide formation Procedure (GP1): Isatoic anhydride derivatives were dissolved in dry DMF (30 mL) and treated with the corresponding amine (5 equiv) or a mixture of the amine hydrochloride (5 equiv) and triethylamine (5 equiv). The mixture was heated to 40–120 °C (depending on the amine) for 3–6 h. For workup, the mixture was poured into water (100 mL) and the product was extracted with EtOAc (5 × 100 mL). The combined organic layers were washed with brine (50 mL), dried over Na_2SO_4 and evaporated to dryness. The crude product was purified by column chromatography to obtain the benzamide derivatives.

General cyclization procedure (GP2): Benzamide derivatives were dissolved in glacial acetic acid (20 mL). The mixture was treated with the corresponding aldehyde (1.2 equiv) and

heated to 70 °C for 1–4 h. Then the mixture was poured onto ice water (20 mL), basified with a NaOH solution (2 M) and the pH was adjusted to 9 with sat. NH_4Cl solution. The product was extracted with EtOAc (3 × 40 mL), the combined organic layers were washed with brine (30 mL), dried over Na_2SO_4 and the solvent was evaporated under reduced pressure. The crude product was either crystallised or purified by column chromatography to obtain dihydroquinazolinones.

General reduction procedure (GP3): Dihydroquinazolinones were dissolved in dry THF (30 mL) at 0 °C and $LiAlH_4$ (4 equiv) was added. The mixture was allowed to reach rt and was then heated to reflux temperature for 1–3 h. After cooling to rt, the mixture was poured into ice water (50 mL) followed by the addition of saturated NH_4Cl solution until pH 9. The aqueous phase was then extracted with EtOAc (3 × 80 mL), the combined organic layers were washed with brine (50 mL), dried over Na_2SO_4 and the solvent was evaporated under reduced pressure. The crude product was purified by column chromatography to obtain the corresponding tetrahydroquinazolines.

Synthesis and experimental data

1-Methyl-2*H*-benzo[*d*][1,3]oxazine-2,4(1*H*)-dione (**4**): A solution of isatoic anhydride (7.60 g, 46.6 mmol, 1 equiv) in DMAc (100 mL) was treated with DIPEA (16.0 mL, 93.2 mmol, 2 equiv) and methyl iodide (11.6 mL, 186 mmol, 4 equiv). After stirring for 24 h at 40 °C, ice-cold water (100 mL) was added. The formed suspension was stirred for further 40 min. The precipitated solid was filtered off, washed with water (2 × 15 mL), cyclohexane (3 × 15 mL), and dried in vacuo to obtain 1-methyl-2*H*-benzo[*d*][1,3]oxazine-2,4(1*H*)-dione (**4**, 7.35 g, 89%) as yellow brown solid; **mp** 174-177 °C. **^{1}H NMR** (DMSO-d_6, 400 MHz): δ = 8.01 (dd, J = 7.8, 1.4 Hz, 1H), 7.88 - 7.84 (m, 1H), 7.44 (d, J = 8.4 Hz, 1H), 7.36 - 7.32 (m, 1H), 3.47 (s, 3H) ppm. **^{13}C NMR** (DMSO-d_6, 101 MHz): δ = 159.0, 147.7, 142.2, 137.1, 129.3, 123.5, 114.8, 111.5, 31.6 ppm.

N-Methyl-2-(methylamino)benzamide (**5a**): According to GP1, 1-methyl-2*H*-benzo[*d*][1,3]oxazine-2,4(1*H*)-dione (**4**, 1.50 g, 8.47 mmol, 1 equiv), methylamine hydrochloride (2.86 g, 42.3 mmol, 5 equiv) and triethylamine (5.86 mL, 42.3 mmol, 5 equiv) were used to obtain *N*-methyl-2-(methylamino)benzamide (**5a**, 1.28 g, 92%) after column

chromatography (petroleum ether/EtOAc 1:1) as yellow oil; **^{1}H NMR** ($CDCl_3$, 400 MHz): δ = 7.41 (br, NH), 7.31 - 7.27 (m, 2H), 6.65 - 6.63 (d, *J* = 8.1 Hz, 1H), 6.57 - 6.52 (m, 1H), 6.04 (br, NH), 2.92 (d, *J* = 4.8 Hz, 3H), 2.83 (s, 3H) ppm. **^{13}C NMR** ($CDCl_3$, 101 MHz): δ = 170.8, 150.6, 132.9, 127.3, 115.5, 114.7, 111.3, 29.9, 26.7 ppm.

2-(Methylamino)-*N*-phenylbenzamide (**5b**): According to GP1, 1-methyl-2*H*-benzo[*d*][1,3]oxazine-2,4(1*H*)-dione (**4**, 700 mg, 3.95 mmol, 1 equiv), and aniline (1.80 mL, 19.8 mmol, 5 equiv) were used to obtain 2-(methylamino)-*N*-phenylbenzamide (**5b**, 625 mg, 70%) after column chromatography (petroleum ether/EtOAc 2:1) as pale red solid; **mp** 125-127 °C. **^{1}H NMR** (DMSO-d_6, 400 MHz): δ = 10.05 (s, NH), 7.72 - 7.70 (m, 2H), 7.67 (dd, *J* = 7.8, 1.3 Hz, 1H), 7.37 - 7.29 (m, 3H+NH), 7.10 - 7.06 (m, 1H), 6.68 (d, *J* = 7.4 Hz, 1H), 6.64 (t, *J* = 7.7 Hz, 1H), 2.80 (d, *J* = 5.0 Hz, 3H) ppm. **^{13}C NMR** (DMSO-d_6, 101 MHz): δ = 168.0, 150.0, 139.1, 132.7, 128.8, 128.4 (2C), 123.4, 120.6 (2C), 115.7, 114.0, 110.6, 29.4 ppm.

N-Isopropyl-2-(methylamino)benzamide (**5c**): According to GP1, 1-methyl-2*H*-benzo[*d*][1,3]oxazine-2,4(1*H*)-dione (**4**, 700 mg, 3.95 mmol, 1 equiv) and isopropylamine (1.69 mL, 19.8 mmol, 5 equiv) were used to obtain *N*-isopropyl-2-(methylamino)benzamide (**5c**, 682 mg, 90%) after column chromatography (petroleum ether/EtOAc 2:1) as white solid; **mp** 119-121 °C. **^{1}H NMR** (DMSO-d_6, 400 MHz): δ = 8.00 (d, *J* = 7.6 Hz, NH), 7.57 (q, *J* = 4.9 Hz, NH), 7.51 (dd, *J* = 7.8, 1.6 Hz, 1H), 7.28 - 7.25 (m, 1H), 6.60 (dd, *J* = 8.4, 0.8 Hz, 1H), 6.56 - 6.52 (m, 1H), 4.12 - 4.00 (m, 1H), 2.76 (d, *J* = 5.1 Hz, 3H), 1.14 (d, *J* = 6.6 Hz, 6H) ppm. **^{13}C NMR** (DMSO-d_6, 101 MHz): δ = 168.2, 149.9, 132.0, 128.3, 115.6, 113.8, 110.3, 40.4, 29.2, 22.3 (2C) ppm.

2-(Methylamino)-*N*-propylbenzamide (**5d**): According to GP1, 1-methyl-2*H*-benzo[*d*][1,3]oxazine-2,4(1*H*)-dione (**4**, 700 mg, 3.95 mmol, 1 equiv) and *n*-propylamine (973 µL, 11.9 mmol, 3 equiv) were used to obtain 2-(methylamino)-*N*-propylbenzamide (**5d**, 759 mg, 97%) after column chromatography (petroleum ether/EtOAc 1:2) as pale yellow solid; **mp** 57-59 °C. **^{1}H NMR** ($CDCl_3$, 400 MHz): δ = 7.40 (br, NH), 7.31 - 7.29 (m, 1H), 7.29 - 7.27 (m, 1H), 6.64 (dd, *J* = 8.6, 0.7 Hz, 1H), 6.58 - 6.54 (m, 1H), 6.01 (br, NH), 3.37 - 3.32 (m, 2H), 2.83 (d, *J* = 4.8 Hz, 3H), 1.60 (sextet, *J* = 7.2 Hz, 2H), 0.96 (t, *J* = 7.4 Hz, 3H)

ppm. **^{13}C NMR** ($CDCl_3$, 101 MHz): δ = 170.1, 150.7, 132.9, 127.2, 115.7, 114.6, 111.3, 41.6, 29.9, 23.2, 11.7 ppm.

1,3-Dimethyl-2-phenyl-2,3-dihydroquinazolin-4(1*H*)-one (**6a**): According to GP2, *N*-methyl-2-(methylamino)benzamide (**5a**, 565 mg, 3.44 mmol, 1 equiv) and benzaldehyde (417 µL, 4.13 mmol, 1.2 equiv) were used to obtain 1,3-dimethyl-2-phenyl-2,3-dihydroquinazolin-4(1*H*)-one (**6a**, 628 mg, 73%) after column chromatography (petroleum ether/EtOAc 2:1) as white solid; **mp** 139-142 °C. **^{1}H NMR** (DMSO-d_6, 400 MHz): δ = 7.77 (dd, *J* = 7.6, 1.4 Hz, 1H), 7.37 - 7.34 (m, 1H), 7.33 - 7.31 (m, 3H), 7.23 - 7.18 (m, 2H), 6.78 (dt, *J* = 7.6, 0.8 Hz, 1H), 6.59 (d, *J* = 8.1 Hz, 1H), 5.78 (s, 1H), 2.88 (s, 3H), 2.80 (s, 3H) ppm. **^{13}C NMR** (DMSO-d_6, 101 MHz): δ = 161.6, 146.2, 137.0, 133.7, 128.9, 128.7 (2C), 127.5, 126.0 (2C), 117.3, 115.7, 111.8, 78.3, 34.9, 31.8 ppm. **ESI-MS**: *m/z* calcd: 252.1, found: 253.1 $[M+H]^+$. **HPLC**: 98%.

2-(4-(*tert*-Butyl)phenyl)-1,3-dimethyl-2,3-dihydroquinazolin-4(1*H*)-one (**6b**): According to GP2, *N*-methyl-2-(methylamino)benzamide (**5a**, 200 mg, 1.22 mmol, 1 equiv) and 4-(*tert*-butyl)benzaldehyde (245 µL, 1.45 mmol, 1.2equiv) were used to obtain 2-(4-(*tert*-butyl)phenyl)-1,3-dimethyl-2,3-dihydroquinazolin-4(1*H*)-one (**6b**, 329 mg, 88%) after column chromatography (petroleum ether/EtOAc 3:1) as white solid; **mp** 126-129 °C. ^{1}H NMR (400 MHz, DMSO-d_6): δ = 7.76 (dd, *J* = 7.6, 1.6 Hz, 1H), 7.41 - 7.24 (m, 3H), 7.13 (d, *J* = 8.3 Hz, 2H), 6.78 (t, *J* = 7.1 Hz, 1H), 6.60 (d, *J* = 8.2 Hz, 1H), 5.75 (s, 1H), 2.88 (s, 3H), 2.81 (s, 3H), 1.23 (s, 9H) ppm. ^{13}C NMR (101 MHz, DMSO): δ = 162.1, 151.7, 146.8, 134.6, 134.2, 127.9, 126.3 (2C), 126.0 (2C), 117.8, 116.3, 112.4, 78.5, 35.5, 34.8, 32.4, 31.5 (3C) ppm.

1,3-Dimethyl-2-(*p*-tolyl)-2,3-dihydroquinazolin-4(1*H*)-one (**6c**): According to GP2, *N*-methyl-2-(methylamino)benzamide (**5a**, 200 mg, 1.22 mmol, 1 equiv) and 4-methylbenzaldehyde (175 µL, 1.45 mmol, 1.2equiv) were used to obtain 1,3-dimethyl-2-(*p*-tolyl)-2,3-dihydroquinazolin-4(1*H*)-one (**6c**, 287 mg, 88%) after column chromatography (petroleum ether/EtOAc 3:1) as white solid; **mp** 133-136 °C. ^{1}H NMR (400 MHz, DMSO-d_6): δ = 7.76 (dd, *J* = 7.6, 1.7 Hz, 1H), 7.37 - 7.28 (m, 1H), 7.16 - 7.05 (m, *J* = 8.0 Hz, 4H), 6.78 (t, *J* = 7.4 Hz, 1H), 6.58 (d, *J* = 8.2 Hz, 1H), 5.73 (s, 1H), 2.87 (s, 3H), 2.78 (s, 3H), 2.25 (s,

3H) ppm. ^{13}C NMR (101 MHz, DMSO-d_6): δ = 162.1, 146.7, 138.8, 134.5, 134.2, 129.8 (2C), 127.9, 126.5 (2C), 117.8, 116.2, 112.3, 78.7, 35.3, 32.3, 21.2 ppm.

2-(4-Fluorophenyl)-1,3-dimethyl-2,3-dihydroquinazolin-4(1*H*)-one (**6d**): According to GP2, *N*-methyl-2-(methylamino)benzamide (**5a**, 200 mg, 1.22 mmol, 1 equiv) and 4-fluorobenzaldehyde (157 µL, 1.45 mmol, 1.2 equiv) were used to obtain 2-(4-fluorophenyl)-1,3-dimethyl-2,3-dihydroquinazolin-4(1*H*)-one (**6d**, 209 mg, 64%) after crystallization from a mixture of petroleum ether/DCM as white solid; **mp** 134-138 °C. ^{1}H NMR (400 MHz, DMSO-d_6): δ = 7.77 (dd, *J* = 7.6, 1.6 Hz, 1H), 7.36 (ddd, *J* = 8.3, 7.3, 1.7 Hz, 1H), 7.28 - 7.21 (m, 2H), 7.21 - 7.13 (m, 2H), 6.79 (td, *J* = 7.5, 0.9 Hz, 1H), 6.60 (d, *J* = 8.2 Hz, 1H), 5.81 (s, 1H), 2.87 (s, 3H), 2.79 (s, 3H) ppm. ^{13}C NMR (101 MHz, DMSO-d_6): δ = 162.8 (d, *J* = 244.9 Hz), 162.0, 146.6, 134.3, 133.8 (d, *J* = 3.1 Hz), 128.7 (d, *J* = 8.4 Hz, 2C), 128.0, 118.0, 116.1 (d, *J* = 21.6 Hz, 2C), 116.1, 112.5, 78.0, 35.3, 32.3 ppm.

1,3-Dimethyl-2-(4-(trifluoromethyl)phenyl)-2,3-dihydroquinazolin-4(1*H*)-one (**6e**):
According to GP2, *N*-methyl-2-(methylamino)benzamide (**5a**, 200 mg, 1.22 mmol, 1 equiv) and 4-(trifluoromethyl)benzaldehyde (200 µL, 1.46 mmol, 1.2 equiv) were used to obtain 1,3-dimethyl-2-(4-(trifluoromethyl)phenyl)-2,3-dihydroquinazolin-4(1*H*)-one (**6e**, 354 mg, 91%) after column chromatography (petroleum ether/EtOAc 1:1) as white solid; **mp** 130-132 °C. **^{1}H NMR** ($CDCl_3$, 400 MHz): δ = 8.01 - 7.99 (m, 1H), 7.54 (d, *J* = 8.1 Hz, 2H), 7.36 - 7.30 (m, 3H), 6.86 (dt, *J* = 7.6, 0.8 Hz, 1H), 6.48 (d, *J* = 8.3 Hz, 1H), 5.45 (s, 1H), 2.99 (s, 3H), 2.81 (s, 3H) ppm. **^{13}C NMR** ($CDCl_3$, 101 MHz): δ = 162.6, 146.0, 140.9, 134.2, 131.7 (q, *J* = 32.8 Hz), 128.9, 126.9 (2C), 126.2 (q, *J* = 3.8 Hz, 2C), 124.0 (q, *J* = 273.2 Hz), 118.8, 116.3, 112.0, 80.5, 35.8, 32.7 ppm.

2-(2,6-Dichlorophenyl)-1,3-dimethyl-2,3-dihydroquinazolin-4(1*H*)-one (**6f**): According to GP2, *N*-methyl-2-(methylamino)benzamide (**5a**, 400 mg, 2.44 mmol, 1 equiv) and 2,6-dichlorobenzaldehyde (512 mg, 2.93 mmol, 1.2 equiv) were used to obtain 2-(2,6-dichlorophenyl)-1,3-dimethyl-2,3-dihydroquinazolin-4(1*H*)-one (**6f**, 571 mg, 73%) after column chromatography (petroleum ether/EtOAc 2:1) as white solid; **mp** 195-201 °C. ^{1}H NMR (400 MHz, $CDCl_3$): δ = 7.92 (dd, *J* = 7.7, 1.6 Hz, 1H), 7.32 - 7.28 (m, 2H), 7.26 (ddd, *J* = 8.4, 7.3, 1.7 Hz, 1H), 7.21 - 7.14 (m, 1H), 6.78 (s, 1H), 6.67 (td, *J* = 7.6, 0.9 Hz, 1H), 6.43 (d, *J* = 8.3 Hz, 1H), 2.72 (s, 3H), 2.62 (s, 3H) ppm. ^{13}C NMR (101 MHz, $CDCl_3$):

δ = 161.9, 146.5, 136.1 (2C), 133.8, 132.3, 130.4, 128.3, 116.7, 113.6, 109.0, 76.0, 33.7, 30.7 ppm.

1-Methyl-2-phenyl-3-propyl-2,3-dihydroquinazolin-4(1*H*)-one (**7a**): According to GP2, 2-(methylamino)-*N*-propylbenzamide (**5d**, 672 mg, 3.50 mmol, 1 equiv) and benzaldehyde (424 µL, 4.19 mmol, 1.2equiv) were used to obtain 1-methyl-2-phenyl-3-propyl-2,3-dihydroquinazolin-4(1*H*)-one (**7a**, 649 mg, 66%) after column chromatography (petroleum ether/EtOAc 3:1) as white solid; **mp** 176-180 °C. **^{1}H NMR** (DMSO-d_6, 400 MHz): δ = 7.77 (dd, *J* = 7.6, 1.6 Hz, 1H), 7.35 - 7.29 (m, 4H), 7.25 - 7.21 (m, 2H), 6.79 (dt, *J* = 7.5, 0.9 Hz, 1H), 6.57 (d, *J* = 8.1 Hz, 1H), 5.78 (s, 1H), 3.79 - 3.72 (m, 1H), 2.82 (s, 3H), 2.79 - 2.72 (m, 1H), 1.67 - 1.54 (m, 1H), 1.54 - 1.41 (m, 1H), 0.84 (t, *J* = 7.4 Hz, 3H) ppm. **^{13}C NMR** (DMSO-d_6, 101 MHz): δ = 161.3, 146.1, 137.7, 133.6, 128.8, 128.6 (2C), 127.5, 126.2 (2C), 117.5, 116.4, 112.1, 76.5, 45.9, 35.0, 20.5, 11.2 ppm.

3-Isopropyl-1-methyl-2-phenyl-2,3-dihydroquinazolin-4(1*H*)-one (**7b**): According to GP2, *N*-isopropyl-2-(methylamino)benzamide (**5c**, 657 mg, 3.42 mmol, 1 equiv) and benzaldehyde (414 µL, 4.1 mmol, 1.2 equiv) were used to obtain 3-isopropyl-1-methyl-2-phenyl-2,3-dihydroquinazolin-4(1*H*)-one (**7b**, 820 mg, 86%) after column chromatography (petroleum ether/EtOAc 2:1) as white solid; **mp** 153-155 °C. **^{1}H NMR** (DMSO-d_6, 400 MHz): δ = 7.96 (dd, *J* = 7.6, 1.5 Hz, 1H), 7.49 - 7.45 (m, 1H), 7.45 - 7.40 (m, 5H), 6.97 (dt, *J* = 7.5, 0.8 Hz, 1H), 6.67 (d, *J* = 8.0 Hz, 1H), 6.00 (s, 1H), 4.84 (septet, *J* = 6.9 Hz, 1H), 3.01 (s, 3H), 1.42 (d, *J* = 6.8 Hz, 3H), 1.03 (d, *J* = 6.9 Hz, 3H) ppm. **^{13}C NMR** (DMSO-d_6, 101 MHz): δ = 161.1, 145.7, 138.8, 133.4, 128.4, 128.3 (2C), 127.5, 126.0 (2C), 117.8, 117.7, 112.5, 72.6, 45.2, 35.0, 20.12, 20.11 ppm.

1-Methyl-2,3-diphenyl-2,3-dihydroquinazolin-4(1*H*)-one (**7c**): According to GP2, 2-(methylamino)-*N*-phenylbenzamide (**5b**, 604 mg, 2.67 mmol, 1 equiv) and benzaldehyde (324 µL, 3.20 mmol, 1.2 equiv) were used to obtain 1-methyl-2,3-diphenyl-2,3-dihydroquinazolin-4(1*H*)-one (**7c**, 772 mg, 93%) after column chromatography (petroleum ether/EtOAc 3:1) as white solid; **mp** 138–142 °C. **^{1}H NMR** (DMSO-d_6, 400 MHz): δ = 7.85 (dd, *J* = 7.7, 1.6 Hz, 1H), 7.43 - 7.39 (m, 1H), 7.39 - 7.34 (m, 2H), 7.31 - 7.28 (m, 3H), 7.27 - 7.22 (m, 5H), 6.85 (dt, *J* = 7.6, 0.7 Hz, 1H), 6.67 (d, *J* = 8.0 Hz, 1H), 6.22 (s, 1H), 2.97 (s, 3H) ppm. **^{13}C NMR** (DMSO-d_6, 101 MHz): δ = 161.2, 146.3, 140.5, 137.5, 134.2, 128.8 (2C),

128.7, 128.6 (2C), 128.1, 126.53 (2C), 126.48, 126.2 (2C), 117.9, 116.7, 112.8, 79.0, 35.5 ppm.

1,3-Dimethyl-2-phenyl-1,2,3,4-tetrahydroquinazoline (**8a**): According to GP3, starting from 1,3-dimethyl-2-phenyl-2,3-dihydroquinazolin-4(1*H*)-one (**6a**, 250 mg, 0.99mmol, 1 equiv) the title compound 1,3-dimethyl-2-phenyl-1,2,3,4-tetrahydroquinazoline (**8a**, 208 mg, 89%) was obtained after column chromatography (petroleum ether/EtOAc 2:1) as brown solid; **mp** 94-96 °C. **^{1}H NMR** ($CDCl_3$, 400 MHz): δ = 7.30 - 7.19 (m, 3H), 7.19 - 7.10 (m, 3H), 6.80 (d, *J* = 7.3 Hz, 1H), 6.61 - 6.55 (m, 2H), 4.79 (s, 1H), 3.71 (d, *J* = 16.0 Hz, 1H), 3.30 (d, *J* = 16.0 Hz, 1H), 2.88 (s, 3H), 2.44 (s, 3H) ppm. **^{13}C NMR** ($CDCl_3$, 101 MHz): δ = 143.8, 141.0, 128.6 (2C), 128.02, 127.95, 127.3 (3C), 118.2, 116.0, 109.0, 82.3, 49.5, 42.5, 36.9 ppm. **ESI-MS**: *m/z* calcd: 238.2, found: 239.1 $[M+H]^+$. **HPLC**: 99%.

2-(4-(*tert*-Butyl)phenyl)-1,3-dimethyl-1,2,3,4-tetrahydroquinazoline (**8b**): According to GP3, starting from 2-(4-(*tert*-butyl)phenyl)-1,3-dimethyl-2,3-dihydroquinazolin-4(1*H*)-one (**6b**) (309 mg, 1.00 mmol, 1 equiv) the title compound 2-(4-(*tert*-butyl)phenyl)-1,3-dimethyl-1,2,3,4-tetrahydroquinazoline (**8b**, 222 mg, 75%) was obtained after column chromatography (petroleum ether/EtOAc 4:1) as white solid; **mp** 96-99 °C. ^{1}H NMR (400 MHz, DMSO-d_6): δ= 7.41 - 7.34 (m, 2H), 7.20 - 7.07 (m, 3H), 6.87 (d, *J* = 6.5 Hz, 1H), 6.70 (d, *J* = 7.8 Hz, 1H), 6.59 (td, *J* = 7.3, 1.0 Hz, 1H), 4.98 (s, 1H), 3.60 (d, *J* = 16.1 Hz, 1H), 3.35 (d, *J* = 14.8 Hz, 1H), 2.96 (s, 3H), 2.44 (s, 3H), 1.30 (s, 9H) ppm.^{13}C NMR (101 MHz, DMSO-d_6): δ = 150.2, 144.0, 138.5, 128.1, 127.1, 126.9 (2C), 125.4 (2C), 118.0, 115.7, 109.2, 80.9, 49.1, 42.0, 37.0, 34.7, 31.6 (3C) ppm. **ESI-MS**: *m/z* calcd: 294.2, found: 295.1 $[M+H]^+$. **HPLC**: 100%.

1,3-Dimethyl-2-(*p*-tolyl)-1,2,3,4-tetrahydroquinazoline (**8c**): According to GP3, starting from 1,3-dimethyl-2-(*p*-tolyl)-2,3-dihydroquinazolin-4(1*H*)-one (**6c**, 269 mg, 1.01 mmol, 1 equiv) the title compound 1,3-dimethyl-2-(*p*-tolyl)-1,2,3,4-tetrahydroquinazoline (**8c**, 203 mg, 80%) was obtained after column chromatography (petroleum ether/EtOAc 4:1) as yellow solid; **mp** 34-36 °C. ^{1}H NMR (400 MHz, DMSO-d_6): δ = 7.15 - 7.06 (m, 3H), 7.05 - 7.00 (m, *J* = 8.0 Hz, 2H), 6.82 (d, *J* = 7.1 Hz, 1H), 6.65 (d, *J* = 7.9 Hz, 1H), 6.54 (td, *J* = 7.3, 1.0 Hz, 1H), 4.91 (s, 1H), 3.55 (d, *J* = 16.0 Hz, 1H), 3.31 (d, *J* = 16.3 Hz, 1H), 2.88 (s, 3H), 2.37 (s, 3H), 2.26 (s, 3H) ppm.^{13}C NMR (101 MHz, DMSO-d_6): δ = 144.0, 138.4, 137.1, 129.2 (2C), 128.1,

127.2 (2C), 127.1, 118.1, 115.7, 109.2, 81.0, 49.1, 42.0, 36.8, 21.1 ppm. **ESI-MS**: *m/z* calcd: 252.2, found: 253.1 $[M+H]^+$. **HPLC**: 99%.

2-(4-Fluorophenyl)-1,3-dimethyl-1,2,3,4-tetrahydroquinazoline (**8d**): According to GP3, starting from 2-(4-fluorophenyl)-1,3-dimethyl-2,3-dihydroquinazolin-4(1*H*)-one (**8c**, 200 mg, 0.74 mmol, 1 equiv) the title compound 2-(4-fluorophenyl)-1,3-dimethyl-1,2,3,4-tetrahydroquinazoline (**8d**, 94 mg, 50%) was obtained after column chromatography (petroleum ether/EtOAc 2:1) as yellow oil; ^{1}H NMR (400 MHz, DMSO-d_6): δ = 7.21 - 7.03 (m, 5H), 6.84 (d, *J* = 7.2 Hz, 1H), 6.67 (d, *J* = 8.1 Hz, 1H), 6.56 (td, *J* = 7.3, 1.0 Hz, 1H), 4.98 (s, 1H), 3.53 (d, *J* = 16.2 Hz, 1H), 3.33 (d, *J* = 12.3 Hz, 1H), 2.91 (s, 3H), 2.38 (s, 3H) ppm.^{13}C NMR (101 MHz, DMSO-d_6): δ = 161.9 (d, *J* = 243.1 Hz), 143.7, 137.6 (d, *J* = 2.9 Hz), 129.2 (d, *J* = 8.2 Hz, 2C), 128.1, 127.2, 118.0, 115.5 (d, *J* = 21.3 Hz, 2C), 115.4, 109.3, 80.4, 48.9, 42.0, 36.9 ppm. **ESI-MS**: *m/z* calcd: 256.1, found: 257.1 $[M+H]^+$. **HPLC**: 96%.

1,3-Dimethyl-2-(4-(trifluoromethyl)phenyl)-1,2,3,4-tetrahydroquinazoline (**8e**): According to GP3, starting from 1,3-dimethyl-2-(4-(trifluoromethyl)phenyl)-2,3-dihydroquinazolin-4(1*H*)-one (**6e**, 337 mg, 1.05 mmol, 1 equiv) the title compound 1,3-dimethyl-2-(4-(trifluoromethyl)phenyl)-1,2,3,4-tetrahydroquinazoline (**8e**, 224 mg, 70%) was obtained after column chromatography (petroleum ether/EtOAc 4:1) as clear oil; **^{1}H NMR** ($CDCl_3$, 400 MHz): δ = 7.53 , (d, *J* = 8.1 Hz, 2H), 7.35 - 7.33 (m, 2H), 7.19 (m, 1H), 6.88 - 6.86 (m, 1H), 6.68 (d, *J* = 8.2 Hz, 1H), 6.66 (dt, *J* = 7.3, 1.0 Hz, 1H), 4.88 (s, 1H), 3.70 (d, 16.2 Hz, 1H), 3.39 - 3.35 (m, 1H), 2.96 (s, 3H), 2.51 (s, 3H) ppm. **^{13}C NMR** ($CDCl_3$, 101 MHz): δ = 145.2, 143.4, 130.2 (q, *J* = 32.4 Hz, 2C), 128.2, 127.7 (2C), 127.4, 125.6 (q, *J* = 3.8 Hz), 124.4 (q, *J* = 273.7 Hz), 118.0, 116.5, 109.3, 81.9, 49.4, 42.6, 37.1 ppm. **ESI-MS**: *m/z* calcd: 306.1, found: 307.0 $[M+H]^+$. **HPLC**: 97%.

2-(2,6-Dichlorophenyl)-1,3-dimethyl-1,2,3,4-tetrahydroquinazoline (**8f**): According to GP3, starting from 2-(2,6-dichlorophenyl)-1,3-dimethyl-2,3-dihydroquinazolin-4(1*H*)-one (**6f**) (520 mg, 1.63 mmol, 1 equiv) the title compound 2-(2,6-dichlorophenyl)-1,3-dimethyl-1,2,3,4-tetrahydroquinazoline (**8f**, 386 mg, 78%) was obtained after column chromatography (petroleum ether/EtOAc 5:1) as white solid; **mp** 78-79 °C. ^{1}H NMR (400 MHz, $CDCl_3$): δ = 7.24 (br, 2H), 7.14 - 7.04 (m, 2H), 6.89 (d, *J* = 7.2 Hz, 1H), 6.57 (td, *J* = 7.3, 1.0 Hz, 1H), 6.49 (d, *J* = 8.1 Hz, 1H), 5.40 (d, *J* = 0.9 Hz, 1H), 3.83 (d, *J* = 15.0 Hz, 1H), 3.41 (d, *J* = 15.0

Hz, 1H), 2.68 (s, 3H), 2.36 (s, 3H) ppm.^{13}C NMR (101 MHz, $CDCl_3$): δ = 144.2, 135.2, 129.0, 128.0, 126.5, 119.3, 115.7, 109.0, 79.9, 52.6, 42.2, 34.7 ppm. **ESI-MS**: *m/z* calcd: 306.1, found: 307.0 $[M+H]^+$. **HPLC**: 99%.

1-Methyl-2-phenyl-3-propyl-1,2,3,4-tetrahydroquinazoline (**9a**): According to GP3, starting from 1-methyl-2-phenyl-3-propyl-2,3-dihydroquinazolin-4(1*H*)-one (**7a**, 350 mg, 1.25 mmol, 1 equiv) the title compound 1-methyl-2-phenyl-3-propyl-1,2,3,4-tetrahydroquinazoline (**9a**, 277 mg, 84%) was obtained after column chromatography (petroleum ether/EtOAc 3:1) as beige solid; **mp**: 46-48 °C. ^{1}**H NMR** ($CDCl_3$, 400 MHz): δ = 7.24 - 7.20 (m, 2H), 7.19 - 7.13 (m, 3H), 7.13 - 7.09 (m, 1H), 6.79 (d, *J* = 7.2 Hz, 1H), 6.59 - 6.53 (m, 2H), 4.90 (s, 1H), 3.68 (d, *J* = 16.3 Hz, 1H), 3.37 - 3.32 (m, 1H), 2.90 (s, 3H), 2.57 - 2.42 (m, 2H), 1.56 (sextet, *J* = 7.3 Hz, 2H), 0.89 (t, *J* = 7.3 Hz, 3H) ppm. ^{13}**C NMR** ($CDCl_3$, 101 MHz): δ = 144.2, 141.6, 128.6 (2C), 127.9, 127.7, 127.24 (2C), 127.16, 118.6, 115.7, 108.7, 80.4, 55.6, 47.6, 37.0, 21.6, 12.1 ppm. **ESI-MS**: *m/z* calcd: 266.2, found: 267.2 $[M+H]^+$. **HPLC**: 100%.

3-Isopropyl-1-methyl-2-phenyl-1,2,3,4-tetrahydroquinazoline (**9b**): According to GP3, starting from 3-isopropyl-1-methyl-2-phenyl-2,3-dihydroquinazolin-4(1*H*)-one (**7b**, 300 mg, 1.07 mmol, 1 equiv) the title compound 3-isopropyl-1-methyl-2-phenyl-1,2,3,4-tetrahydroquinazoline (**9b**, 247 mg, 87%) was obtained after column chromatography (petroleum ether/EtOAc 4:1) as yellow solid; **mp**: 40-42 °C. ^{1}**H NMR** ($CDCl_3$, 400 MHz): δ = 7.31 - 7.28 (m, 1H), 7.27 - 7.26 (m, 1H), 7.24 - 7.20 (m, 3H), 7.18 - 7.13 (m, 1H), 6.85 - 6.83 (m, 1H), 6.62 - 6.58 (m, 2H), 5.19 (s, 1H), 3.73 (d, *J* = 16.6 Hz, 1H), 3.62 (d, *J* = 16.6, 1H), 2.94 (s, 3H), 2.87 (septet, *J* = 6.3 Hz, 1H), 1.20 (d, *J* = 6.3 Hz, 3H), 1.23 (d, *J* = 6.4 Hz, 3H) ppm. ^{13}**C NMR** ($CDCl_3$, 101 MHz): δ = 144.9, 142.3, 128.6 (2C), 127.9, 127.5, 127.2 (2C), 126.6, 119.5, 115.6, 108.5, 77.6, 50.0, 44.7, 36.6, 22.1, 21.7 ppm. **ESI-MS**: *m/z* calcd: 266.2, found: 267.1 $[M+H]^+$. **HPLC**: 99%.

1-Methyl-2,3-diphenyl-1,2,3,4-tetrahydroquinazoline (**9c**): According to GP3, starting from 1-methyl-2,3-diphenyl-2,3-dihydroquinazolin-4(1*H*)-one (**7c**, 310 mg, 0.99mmol, 1 equiv) the title compound 1-methyl-2,3-diphenyl-1,2,3,4-tetrahydroquinazoline (**9c**, 178 mg, 61%) was obtained after crystallization from petroleum ether/DCM as pale yellow solid; **mp** 132-134 °C. ^{1}**H NMR** ($CDCl_3$, 400 MHz): δ = 7.25 - 7.22 (m, 1H), 7.22 - 7.17 (m, 4H), 7.17 - 7.14 (m, 2H), 7.10 - 7.06 (m, 1H), 7.00 - 6.97 (m, 2H), 6.86 - 6.84 (m, 1H), 6.80 (tt, *J* = 7.3, 1.0 Hz, 1H), 6.60 - 6.56 (m, 2H), 5.77 (s, 1H), 4.16 (d, *J* = 16.4 Hz, 1H), 4.10 (d, *J* = 16.2 Hz,

1H), 2.96 (s, 3H) ppm. **^{13}C NMR** ($CDCl_3$, 101 MHz): δ = 150.5, 144.4, 140.5, 129.4 (2C), 128.7 (2C), 128.1, 128.0, 127.1 (2C), 126.4, 120.8, 120.0, 118.6 (2C), 116.6, 110.2, 78.8, 46.7, 37.2 ppm. **ESI-MS**: *m/z* calcd: 300.2, found: 301.1 $[M+H]^+$. **HPLC**: 99%.

<u>2-Amino-*N*-methylbenzamide (**10**):</u> According to GP1, isatoic anhydride (1.00 g, 6.13 mmol, 1 equiv), methylamine hydrochloride (2.07 g, 30.7 mmol, 5 equiv) and triethylamine (4.25 mL, 30.7 mmol, 5 equiv) were used to obtain 2-amino-*N*-methylbenzamide (**10**, 862 mg, 94%) after column chromatography (petroleum ether/EtOAc 1:1) as white solid; **mp**: 80-82 °C. **^{1}H NMR** ($CDCl_3$, 400 MHz): δ = 7.27 (dd, *J* = 7.8, 1.48 Hz, 1H), 7.20 - 7.16 (m, 1H), 6.66 (dd, *J* = 8.2, 0.8 Hz, 1H), 6.64 - 6.60 (m, 1H), 6.02 (br, NH), 5.47 (br, NH_2), 2.95 (d, *J* = 4.8 Hz, 3H) ppm. **^{13}C NMR** ($CDCl_3$, 101 MHz): δ = 170.2, 148.8, 132.4, 127.2, 117.5, 116.8, 116.5, 26.7 ppm.

<u>3-Methyl-2-phenyl-2,3-dihydroquinazolin-4(1*H*)-one (**11**):</u> According to GP2, 2-amino-*N*-methylbenzamide (**10**, 529 mg, 3.52 mmol, 1 equiv) and benzaldehyde (436 μL, 4.23 mmol, 1.2 equiv) were used to obtain 3-methyl-2-phenyl-2,3-dihydroquinazolin-4(1*H*)-one (**11**, 628 mg, 75%) after crystallization from a mixture of petroleum ether/DCM as white solid; **mp** 165-167 °C. **^{1}H NMR** (DMSO-d_6, 400 MHz): δ = 7.65 (dd, *J* = 7.6, 1.4 Hz, N*H*), 7.39 - 7.35 (m, 2H), 7.34 - 7.30 (m, 4H), 7.22 - 7.18 (m, 1H), 6.67 - 6.62 (m, 2H), 5.82 (d, *J* = 2.3 Hz, 1H), 2.85 (s, 3H) ppm. **^{13}C NMR** (DMSO-d_6, 101 MHz): δ = 162.5, 146.4, 140.6, 133.2, 128.6 (2C), 128.5, 127.3, 126.1 (2C), 116.9, 114.3, 114.0, 72.0, 32.0 ppm.

<u>3-Methyl-2-phenyl-1-propyl-2,3-dihydroquinazolin-4(1*H*)-one (**12a**):</u> Starting from 3-methyl-2-phenyl-2,3-dihydroquinazolin-4(1*H*)-one (**11**, 236 mg, 0.99 mmol, 1 equiv) dissolved in dry DMF (10 mL), *n*-propyl bromide (452 μL, 4.96 mmol, 5 equiv) and *t*-BuOK (557 mg, 4.96 mmol, 5 equiv) were added successively. The reaction mixture was stirred for 16 h at 110 °C. Ice-cold water (30 mL) was then added and the mixture was stirred for further 30 min. The product was extracted with EtOAc (3 × 30 mL), the combined organic layers were washed with brine (30 mL), dried over Na_2SO_4 and the solvent was evaporated under reduced pressure to yield 3-methyl-2-phenyl-1-propyl-2,3-dihydroquinazolin-4(1*H*)-one (**12a**, 200 mg, 72%) after column chromatography (petroleum ether/EtOAc 1:1) as white solid; **mp** 116-119 °C. **^{1}H NMR** ($CDCl_3$, 400 MHz): δ = 7.97 (dd, *J* = 7.7, 1.5 Hz, 1H), 7.32 - 7.24 (m, 4H), 7.23 - 7.20 (m, 2H), 6.80 - 6.76 (m, 1H), 6.57 (d, *J* = 8.2 Hz, 1H), 5.43 (s, 1H), 3.35 - 3.28 (m,

1H), 3.05 - 2.97 (m, 4H), 1.72 - 1.59 (m, 1H), 1.57 - 1.46 (m, 1H), 0.92 (t, *J* = 7.4 Hz, 3H) ppm. **^{13}C NMR** ($CDCl_3$, 101 MHz): δ = 162.8, 146.1, 139.0, 133.7, 129.2, 129.0 (2C), 128.9, 126.5 (2C), 118.0, 116.7, 112.6, 79.6, 51.4, 32.9, 20.7, 11.6 ppm.

2-(Isopropylamino)benzoic acid **(14)**: To a solution of 2-aminobenzoic acid (1 g, 7.3 mmol, 1 equiv) in methanol (20 mL) was added acetone (643 µL, 8.76 mmol, 1.2 equiv). The mixture was heated to reflux temperature and stirred for 5 h. After cooling to room temperature, $NaBH_4$ (333 mg, 8.76 mmol, 1.2 equiv) was added portion wise and stirring was continued for further 3 h. The solvent was then evaporated to dryness and suspended in water. The product was extracted with DCM (3 × 150 mL), the organic layer was dried over Na_2SO_4 and the solvent was removed. The crude product was purified by column chromatography (petroleum ether/EtOAc 4:1) to yield 2-(isopropylamino)benzoic acid (**14**, 794 mg, 61%) as white solid; **mp** 110-112 °C. **^{1}H NMR** (400 MHz, DMSO-d$_6$): δ = 12.52 (br, COOH), 7.78 (dd, *J* = 8.0, 1.6 Hz, 1H), 7.35 (ddd, *J* = 8.7, 7.1, 1.8 Hz, 1H), 6.74 (d, *J* = 8.5 Hz, 1H), 6.52 (ddd, *J* = 8.0, 7.1, 1.1 Hz, 1H), 3.72 (hept, *J* = 6.2 Hz, 1H), 1.19 (d, *J* = 6.3 Hz, 6H) ppm. **^{13}C NMR**(101 MHz, DMSO-d$_6$): δ = 170.5, 150.5, 134.9, 132.3, 114.3, 112.1, 110.2, 43.0, 23.1 (2C) ppm.

2-(Isopropylamino)-*N*-methylbenzamide **(15a)**: A mixture of 2-(isopropylamino)benzoic acid (**14**, 770 mg, 4.3 mmol, 1 equiv), methylamine hydrochloride (1.45 g, 21.5 mmol, 5 equiv), triethylamine (2.98 mL, 21.5 mmol, 5 equiv), EDCI hydrochloride (991 mg, 5.16 mmol, 1.2 equiv) and HOBT (697 mg, 5.16 mmol, 1.2 equiv) in DMF (50 mL) were heated to 70 °C for 14 h. The mixture was then poured into water (150 mL) and extracted with EtOAc (4 × 100 mL). The combined organic layers were washed with brine (100 mL), dried over Na_2SO_4 and the solvent was removed under reduced pressure. The crude product was purified by column chromatography (petroleum ether/EtOAc 4:1) to yield 2-(isopropylamino)-*N*-methylbenzamide (**15a**, 627 mg, 76%) as clear oil; **^{1}H NMR** (400 MHz, $CDCl_3$): δ = 7.66 (br, NH), 7.35 - 7.25 (m, 2H), 6.73 (d, *J* = 8.4 Hz, 1H), 6.62 - 6.47 (m, 1H), 6.12 (br, NH), 3.68 (hept, *J* = 6.3 Hz, 1H), 2.97 (d, *J* = 4.8 Hz, 3H), 1.27 (d, *J* = 6.3 Hz, 6H) ppm. **^{13}C NMR** (101 MHz, $CDCl_3$): δ = 170.6, 148.7, 132.6, 127.4, 115.0, 114.3, 112.3, 43.5, 26.5, 22.7 (2C) ppm.

N-Methyl-2-(phenylamino)benzamide (**15b**): To a solution of 2-(phenylamino)benzoic acid (1.00 g, 4.69 mmol, 1 equiv) in dry DMF (40 mL), 1-HOBt (761 mg, 5.63 mmol, 1.2 equiv), EDCI hydrochloride (1.08 g, 5.63 mmol, 1.2 equiv), methylamine hydrochloride (1.59 g, 23.5 mmol, 4 equiv), and triethylamine (3.25 mL, 23.5 mmol, 5 equiv) were added successively. After stirring for 8 h at ambient temperature, the reaction mixture was poured onto water (60 mL) and extracted with EtOAc (3 × 30 mL). The combined organic layers were washed with a 0.1 M HCl (2 × 20 mL), brine (30 mL), and dried over Na_2SO_4. The solvent was evaporated under reduced pressure and the crude product was purified by column chromatography (petroleum ether/EtOAc 2:1) to obtain the title compound **15b** (584 mg, 56%) as white solid; **mp** 85-87 °C. **^{1}H NMR** ($CDCl_3$, 400 MHz): δ = 9.22 (br, N*H*), 7.32 (dd, *J* = 7.8, 1.6 Hz, 1H), 7.29 - 7.24 (m, 1H), 7.23 - 7.16 (m, 3H), 7.13 - 7.10 (m, 2H), 6.92 (tt, *J* = 7.3, 1.2 Hz, 1H), 6.70 - 6.66 (m, 1H), 6.09 (br, N*H*), 2.91 (d, *J* = 4.9 Hz, 3H) ppm. **^{13}C NMR** ($CDCl_3$, 101 MHz): δ = 170.4, 145.5, 141.8, 132.3, 129.5 (2C), 127.6, 122.5, 120.9 (2C), 118.8, 118.1, 115.7, 26.8 ppm.

1-Isopropyl-3-methyl-2-phenyl-2,3-dihydroquinazolin-4(1*H*)-one (**12b**): According to GP2, 2-(isopropylamino)-*N*-methylbenzamide (**15a**, 600 mg, 3.13 mmol, 1 equiv) and benzaldehyde (379 µL, 3.75 mmol, 1.2 equiv), were used to obtain 1-isopropyl-3-methyl-2-phenyl-2,3-dihydroquinazolin-4(1*H*)-one (**12b**, 648 mg, 74%) after column chromatography (petroleum ether/EtOAc 3:1) as white solid; **mp** 107-109 °C. **^{1}H NMR** (400 MHz, $CDCl_3$): δ = 7.92 - 7.84 (m, 1H), 7.23 (ddd, *J* = 8.3, 7.3, 1.7 Hz, 1H), 7.18 - 7.08 (m, 5H), 6.87 - 6.79 (m, 2H), 5.46 (s, 1H), 3.87 (hept, *J* = 6.7 Hz, 1H), 3.10 (s, 3H), 1.29 (d, *J* = 6.8 Hz, 3H), 1.14 (d, *J* = 6.6 Hz, 3H) ppm. **^{13}C NMR** (101 MHz, $CDCl_3$): δ =163.3, 146.6, 140.2, 132.9, 128.5 (2C), 128.1, 127.9, 126.3 (2C), 121.8, 120.8, 119.6, 71.9, 55.0, 33.1, 21.8, 20.3 ppm.

3-Methyl-1,2-diphenyl-2,3-dihydroquinazolin-4(1*H*)-one (**12c**): According to GP2, *N*-methyl-2-(phenylamino)benzamide (**15b**, 454 mg, 2.01 mmol, 1 equiv) and benzaldehyde (244 µL, 2.41 mmol, 1.2 equiv) were used to obtain 3-methyl-1,2-diphenyl-2,3-dihydroquinazolin-4(1*H*)-one (**12c**, 488 mg, 78%) after crystallization from a mixture of petroleum ether/DCM as white solid; **mp** 158-160 °C. **^{1}H NMR** ($CDCl_3$, 400 MHz): δ = 8.00 - 7.99 (m, 1H), 7.35 - 7.32 (m, 2H), 7.31 - 7.26 (m, 5H), 7.26 - 7.22 (m, 1H), 7.17 - 7.14 (m, 2H), 7.14 - 7.12 (m, 1H), 6.96 - 6.92 (m, 1H), 6.84 - 6.81 (m, 1H), 5.93 (s, 1H), 3.16 (s, 3H) ppm. **^{13}C NMR**

($CDCl_3$, 101 MHz): δ = 162.8, 146.3, 144.1, 139.5, 133.1, 129.9 (2C), 129.0 (2C), 128.9, 128.7, 126.6 (2C), 125.0, 123.5 (2C), 121.4, 120.6, 118.9, 80.2, 34.3 ppm.

3-Methyl-2-phenyl-1-propyl-1,2,3,4-tetrahydroquinazoline (**13a**): According to GP3, starting from 3-methyl-2-phenyl-1-propyl-2,3-dihydroquinazolin-4(1*H*)-one (**12a**, 180 mg, 0.64 mmol, 1 equiv) the title compound 3-methyl-2-phenyl-1-propyl-1,2,3,4-tetrahydroquinazoline (**13a**, 140 mg, 82%) was obtained after column chromatography (petroleum ether/EtOAc 3:1) as grey solid; **mp** 45-46 °C. **^{1}H NMR** ($CDCl_3$, 400 MHz): δ = 7.31 - 7.21 (m, 5H), 7.16 - 7.12 (m, 1H), 6.85 - 6.83 (m, 1H), 6.67 (d, *J* = 8.2 Hz, 1H), 6.59 (dt, *J* = 7.3, 1.0 Hz, 1H), 4.91 (s, 1H), 3.76 (d, *J* = 16.2 Hz, 1H), 3.52 - 3.41 (m, 1H), 3.30 (dd, *J* = 16.2, 1.0 Hz, 1H), 2.97 - 2.85 (m, 1H), 2.51 (s, 3H), 1.74 - 1.54 (m, 2H), 0.92 (t, *J* =7.4 Hz, 3H) ppm. **^{13}C NMR** ($CDCl_3$, 101 MHz): δ = 142.9, 141.6, 128.5 (2C), 127.89, 127.86, 127.7, 127.4 (2C), 117.6, 115.5, 108.9, 80.9, 50.9, 49.2, 42.5, 20.7, 11.6 ppm. **ESI-MS**: *m/z* calcd: 266.2, found: 267.1 $[M+H]^+$. **HPLC**: 99%.

1-Isopropyl-3-methyl-2-phenyl-1,2,3,4-tetrahydroquinazoline (**13b**): According to GP3, starting from 1-isopropyl-3-methyl-2-phenyl-2,3-dihydroquinazolin-4(1*H*)-one (**12b**, 300 mg, 1.07 mmol, 1 equiv) the title compound 1-isopropyl-3-methyl-2-phenyl-1,2,3,4-tetrahydroquinazoline (**13b**, 174 mg, 61%) was obtained after column chromatography (petroleum ether/EtOAc 3:1) as clear oil; **^{1}H NMR** (400 MHz, $CDCl_3$): δ = 7.25 - 7.13 (m, 5H), 7.13 - 7.07 (m, 1H), 6.83 - 6.73 (m, 2H), 6.55 (td, *J* = 7.3, 0.9 Hz, 1H), 5.04 (s, 1H), 4.18 (hept, *J* = 6.6 Hz, 1H), 3.71 (d, *J* = 16.2 Hz, 1H), 3.25 (d, *J* = 16.1 Hz, 1H), 2.40 (s, 3H), 1.20 (d, *J* = 6.5 Hz, 3H), 1.00 (d, *J* = 6.8 Hz, 3H) ppm. **^{13}C NMR** (101 MHz, $CDCl_3$): δ =142.9, 142.3, 128.2 (2C), 127.9, 127.7, 127.5, 127.3 (2C), 117.4, 115.2, 109.3, 74.2, 49.0, 46.6, 42.1, 20.7, 19.9 ppm. **ESI-MS**: *m/z* calcd: 266.2, found: 267.1 $[M+H]^+$. **HPLC**: 94%.

3-Methyl-1,2-diphenyl-1,2,3,4-tetrahydroquinazoline (**13c**): According to GP3, starting from 3-methyl-1,2-diphenyl-2,3-dihydroquinazolin-4(1*H*)- one (**12c**, 200 mg, 0.64mmol, 1 equiv) the title compound 3-methyl-1,2-diphenyl-1,2,3,4-tetrahydroquinazoline (**13c**, 153 mg, 81%) was obtained after column chromatography (petroleum ether/EtOAc 3:1) as pale yellow solid; **mp** 107-109 °C. **^{1}H NMR** ($CDCl_3$, 400 MHz): δ = 7.47 - 7.44 (m, 2H), 7.33 - 7.29 (m, 2H), 7.28 - 7.25 (m, 3H), 7.25 - 7.22 (m, 2H), 7.10 - 7.06 (m, 1H), 7.06 - 7.01 (m, 2H), 6.87 (dd, *J* = 7.5 Hz, 0.6 Hz, 1H), 6.73 (dt, *J* = 7.2, 1.3 Hz, 1H), 5.32 (s, 1H), 3.89 (d, *J* = 16.4 Hz, 1H), 3.47 (d, *J* = 16.7 Hz, 1H), 2.63 (s, 3H) ppm. **^{13}C NMR** ($CDCl_3$, 101 MHz): δ = 147.7, 141.4,

141.1, 129.5 (2C), 128.7 (2C), 127.8 (2C), 127.5 (2C), 127.0, 123.7, 123.4 (2C), 119.7, 118.9, 115.2, 82.4, 49.3, 42.4 ppm. **ESI-MS**: *m/z* calcd: 300.2, found: 301.1 $[M+H]^+$. **HPLC**: 99%.

N-Methyl-2-((methylamino)methyl)aniline (**16**): *N*-methyl-2-(methylamino)benzamide (**5a**, 300 mg, 1.83 mmol, 1 equiv) was dissolved in dry THF (20 mL) and treated with $LiAlH_4$ (278 mg, 7.32 mmol, 4 equiv) under ice cooling. The mixture was heated for 24 h to reflux temperature under argon atmosphere, poured into water (30 mL) and extracted with DCM (3 × 30 mL). The combined organic layers were extracted with a 1 M HCl solution (3 × 30 mL). The aqueous phase was washed with DCM (1 × 30 mL), basified with concentrated NH_3 solution and extracted with DCM (3 × 30 mL). The combined organic layers were dried over Na_2SO_4 and the solvent was evaporated under reduced pressure to obtain *N*-methyl-2-((methylamino)methyl)aniline (**16**, 247 mg, 90%) as a yellow liquid; . **^{1}H NMR** ($CDCl_3$, 400 MHz): δ = 7.13 (td, *J* = 7.7, 1.6 Hz, 1H), 6.97 - 6.92 (m, 1H), 6.58 - 6.52 (m, 2H), 3.65 (s, 2H), 2.76 (s, 3H), 2.33 (s, 3H) ppm.**^{13}C NMR** ($CDCl_3$, 101 MHz): δ = 149.41, 129.53, 128.64, 123.70, 115.99, 109.60, 55.60, 36.07, 30.21 ppm. **ESI-MS**: *m/z* calcd: 150.1, found: 151.2 $[M+H]^+$.

Stability tests

Preparation of phosphate-buffered systems

For the preparation of phosphate-buffered solutions with defined pH values, 0.1 M H_3PO_4 was titrated with a 0.3 M NaOH solution while monitoring the pH value on a Metohm 744 pH Meter (Metrohm GmbH & Co. KG, Filderstadt, Germany). Using this procedure, phosphate-buffered systems were obtained with pH 2, 3, 4, 5, 6, 7, 8 and 12.

Hydrolysis experiments

A calibration curve for each test compound and their respective aldehydes as hydrolysis products was recorded. For stability analyses, 300 μL of a solution of the test compound (333 μg/mL) in acetonitrile was thoroughly mixed with 700 μL of a phosphate buffered solution with defined pH value to give a solution with a final test concentration of 100 μg/mL (a certain amount of acetonitrile is necessary to keep the compound in solution). The reaction mixture was incubated for 1 h before it was directly analysed with HPLC by injecting a volume of 20 μL of the test solution. Analytical HPLC was performed on a system from Shimadzu equipped with a DGU-20A3R controller, LC20AB liquid chromatograph, and a SPD-20A UV/Vis detector. Stationary phase was a Synergi 4U fusion-RP (150 × 4.6 mm) column (Phenomenex, Aschaffenburg, Germany). As mobile phase, water (phase A) and MeOH (phase B) were used with 1 mL/min. (conc. B: 5% → 90% from 0 to 8 min; 90% from 8 to 13 min; 90% → 5% from 13 to 15 min; 5% from 15 to 18 min.). Using the beforehand recorded calibration curves, it was possible to calculate the ration between the intact tetrahydroquinazoline as well as the respective aldehyde as its hydrolysis product. Data were subjected to one way ANOVA followed by Dunnett's multiple comparison post test using GraphPad Prism 4 Software (levels of significance * $p < 0.05$; ** $p < 0.01$; *** $p < 0.001$). Each experiment was performed three times with three independently prepared test solutions.

Kinetic monitoring of the hydrolysis of compound **8a** at different pH values (3, 4 and 5) was followed the above mentioned procedure after t = 0, 18.5, 37, 55.5, 74, 92,5 111, 129.5, 148, 166.5, 185, 203.5, 222, 240.5, 259 and 277.5 min. Due to the fast hydrolysis at pH 2, the time interval was shortened to t = 0, 1, 2, 3, 4, 6, 18.5, 37 and 55.5 min. Calculation of k_2 was performed under assumption of a pseudo-first-order kinetics using

$$Y = (Y_0)*\exp(-k_2*t)$$

With Y = amount of non-hydrolysed test compound (%) in dependency of time and Y_0 = amount of non-hydrolysed test compound (%) at *t* = 0 min. All data were analysed using GraphPad Prism 4 Software.

<u>Aminal condensation experiments:</u>

To proof that acid-mediated hydrolysis of the described tetrahydroquinazolines can be reversed by changing the reaction conditions to basic ones, the synthesis of tetrahydroquinazoline **8a** was performed using *N*-methyl-2-((methylamino)methyl)aniline (**16**) and benzaldehyde:

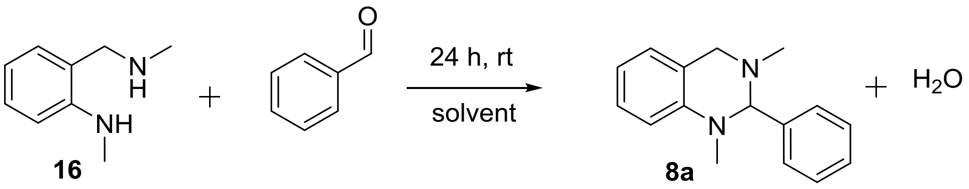

Therefore, *N*-methyl-2-((methylamino)methyl)aniline (**16**, 100 mg, 0.67 mmol, 1 equiv) and benzaldehyde (1.2 equiv) were mixed at room temperature in either a) acetonitrile (1 mL) as reference for the condensation, b) water (1 mL) and c) water/acetonitrile (1:1, 1 mL). After 24 h the reaction mixtures were directly analysed by LC–MS (cf. general part).

Chromatogram for the reaction in water:

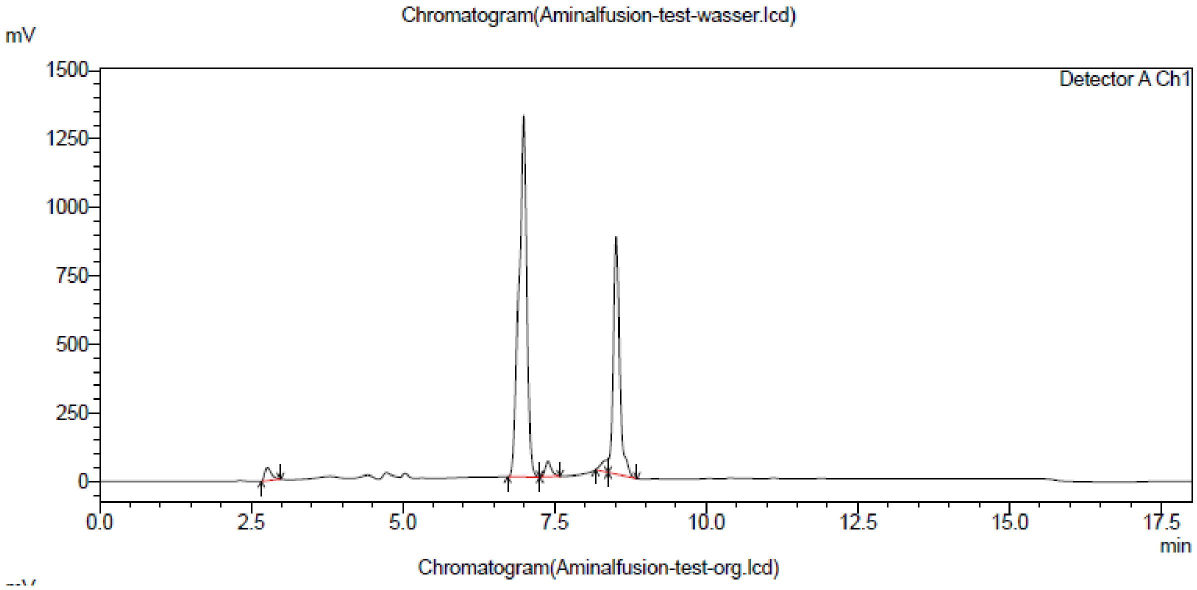

Chromatogram for the reaction in acetonitrile:

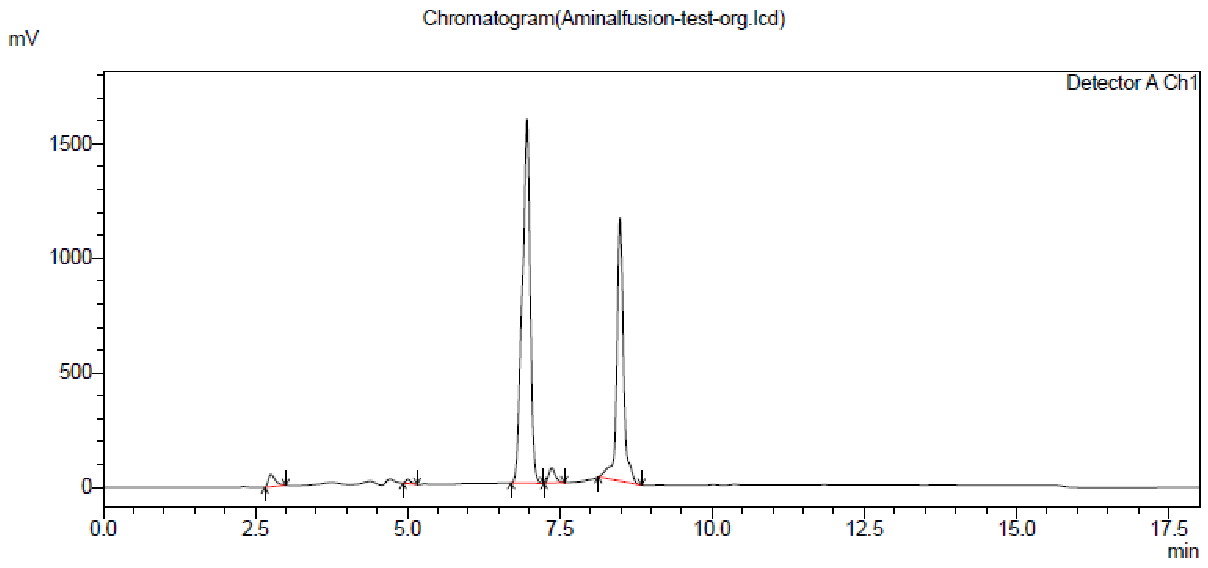

Chromatogram for the reaction in acetonitrile/water:

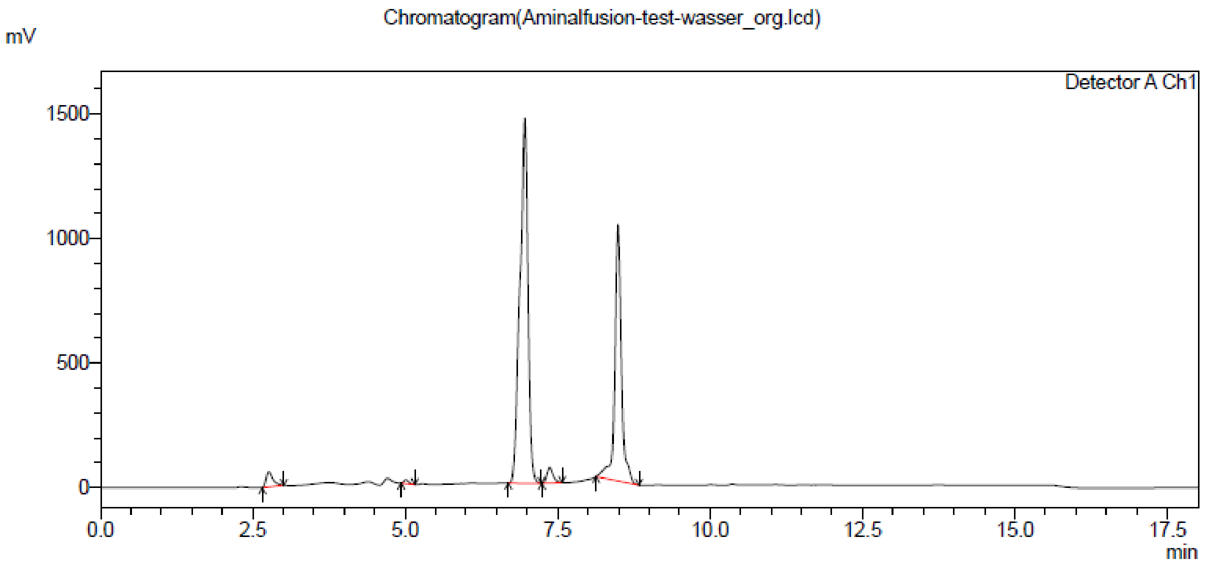

Interestingly, all reactions showed the same result with complete consumption of *N*-methyl-2-((methylamino)methyl)aniline **16** at $t_R \approx 5$ min and benzyldehyde at $t_R = 2.2$ min while forming the tetrahydroquinazoline **8a** ($t_R \approx 7$ min). It is remarkable, that there is another peak at $t_R \approx 8$ min for all cases which could not be identified by mass spectrometry. This peak might be a byproduct from the reaction of benzaldehyde due to the fact, that benzaldehyde is completely consumed although being used in excess. However, these results clearly indicated, that the condensation of diamines and aldehydes is possible in neutral aqueous media, while the hydrolysis is promoted in acidic aqueous media (see main manuscript).

Computational section

Materials and methods

Conformational search: All compounds prior to the conformational search were minimised in MOE[1] using the MMFF94x forcefield[2] in gas phase (ε = 1) and an RMS-gradient of 0.0001 kcal/mol Å.

The systematic search was conducted in Macromodel 10.3 (Schrodinger 2014-1 platform) using the Systematic Pseudo Monte Carlo (SPMC) method[3]. The conformational search was conducted in MMFF94s forcefield[4] and the potential treatment was set according to the Macromodel built-in module for water (ε = 7.8000E+1). The convergence threshold for minimization was set at 0.0001 kcal/mol Å and the maximum iteration limit was set at 25000. Miscellaneous technical characteristics: Maximum number of steps 100000; steps per rotational bond 10000; energy window 478.01 kcal/mol; maximum atom deviation cut-off: 0.01 Å

MM relative ring strain comparison: The transformation of the structures and the potential energy calculation was conducted in the Schrodinger 2014-1 platform environment, using the MMFF94s forcefield. The potential treatment for calculations in water environment was set according to the Macromodel built-in module for water as previously described.

QM single point energy calculations: All tetrahydroquinazoline structures used were results of the aforementioned MM systematic conformational search. All other structures were designed and minimised in Maestro (Schrodinger platform) using the MMFF94s forcefield. The Brønsted acid was simulated by an ammonium cation to facilitate the calculations.

The single point calculations were performed with DFT using the functional B3LYP-D3[5–9] (grid m4[10]) in Turbomole (dscf[11,12]). The basis set used was cc-pVTZ[13,14].

Calculations were performed in water environment (ε = 78.5) and in gas phase. The Conductor-Like Screening Model (COSMO)[15] was used to form the implicit water continuum solvation model. The studied structures were optimised in water and gas phase, respectively.

MoKa[16] calculations

Structure	N1 pK_a	SD	Quality parameter	N3 pK_a	SD	Quality parameter
**8a**	-0,86	0,51	-0,40	6,31	0,60	0,00
**8c**	-0,86	0,51	-0,40	6,31	0,60	0,00
**8d**	-1,00	0,51	-0,40	6,06	0,60	0,00
**8b**	-0,92	0,51	-0,40	6,25	0,60	0,00
**8e**	-0,51	0,51	-0,40	6,66	0,60	0,00
**8f**	-1,00	0,51	-0,40	4,84	0,60	0,00
**9a**	-0,82	0,51	-0,40	6,35	0,60	0,00
**9b**	-0,56	0,51	-0,40	6,61	0,60	0,00
**9c**	8,85	0,51	0,00	2,41	0,51	0,00
**13a**	-0,72	0,51	-0,40	6,45	0,60	0,00
**13b**	-0,69	0,51	-0,40	6,48	0,60	0,00
**13c**	-1,00	0,51	-0,40	6,00	0,60	0,00
**6a**	1,98	0,51	-0,04	NA	NA	NA

Quality parameter (QP): The quality parameter can be used to check whether the compound contains features that MoKa cannot take into account when it predicts the specific pK_a.

QP = 0 indicates that all the chemical groups in the compound have appropriate parameters to calculate the specific pK_a.

QP< or > indicates that the effect of one or more chemical groups on the specific pK_a is unknown. The higher the absolute value of the QP, the higher is the expected effect of these groups on pK_a. The +/− sign before QP specifies whether these groups are expected to shift pK_a upwards or downwards.

QM calculations

Absolute energies of all reactants

Table S1: Absolute energy values.

	B3LYP-D3 water (kcal/mol)	B3LYP-D3 gas (kcal/mol)
H_2O	-47962,92	-47955,99
NH_4^+	-35780,86	-35698,10
NH_3	-35489,63	-35484,54
PhCHO	-216805,79	-216799,60
8a_neutral	-457969,49	-457963,11
8a_N3	-458256,03	-458205,26
8a_N1	-458247,24	-458200,30
8a_N1_N3	-458515,90	-458347,50
N-methyl-2-[(methylamino)methyl]aniline_neutral	-289123,51	-289116,71
N-Methyl-2-[(methylamino)methyl]aniline_N1	-289416,51	-289365,03
N-Methyl-2-[(methylamino)methyl]aniline_N3	-289419,85	-289366,17
N-Methyl-2-[(methylamino)methyl]aniline_N1_N3	-289693,03	-289505,43
13b_neutral	-507287,54	-507281,09
13b_N3	-507574,42	-507524,90
13b_N1	-507564,37	-507520,02
13b_N1_N3	-507832,45	-507672,01
N-Isopropyl-2-[(methylamino)methyl]aniline_neutral	-338449,55	-338444,20
N-Isopropyl-2-[(methylamino)methyl]aniline_N1	-338738,70	-338690,85
N-Isopropyl-2-[(methylamino)methyl]aniline_N3	-338742,54	-338690,41
N-Isopropyl-2-[(methylamino)methyl]aniline_N1_N3	-339015,83	-338836,56

Determining the protonation pattern:

Energy differences between the different protonation patterns for compounds **8a** and **13b** in water revealed that protonation is most likely at the *N*-3 nitrogen.

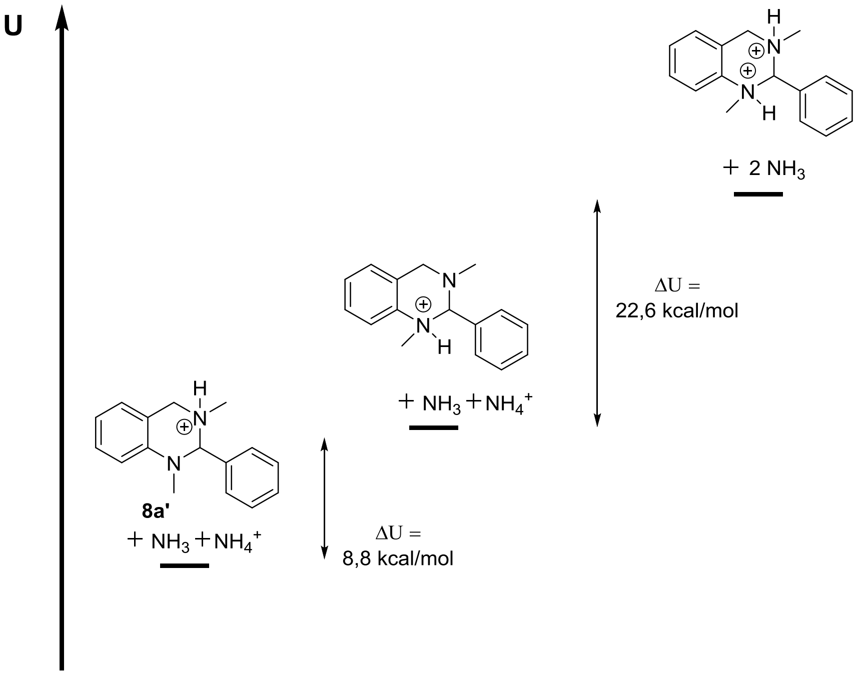

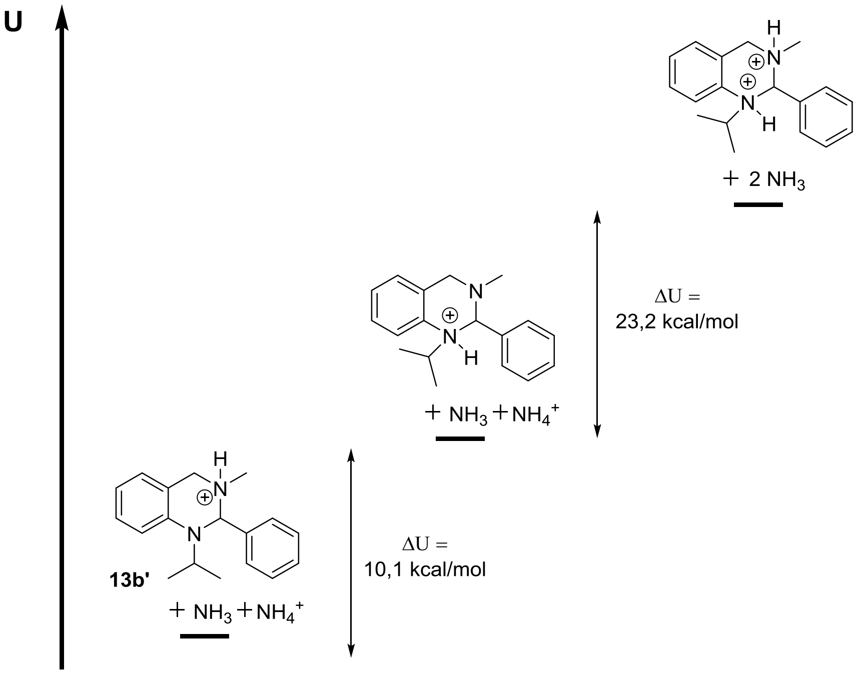

QM summary

The scheme below depicts a reaction pathway. The energy difference ($\Delta U = U_2 - U_1$) between different reaction states (**A** to **H**) for compounds **8a** and **13b** is presented in Table 2. $\Delta U < 0$ indicates that the respective reaction is spontaneous (exothermic) while $\Delta U > 0$ indicates that it is not (endothermic).The energy of each state is calculated from the absolute energies of the molecules that comprise it, which are presented in Table S1.

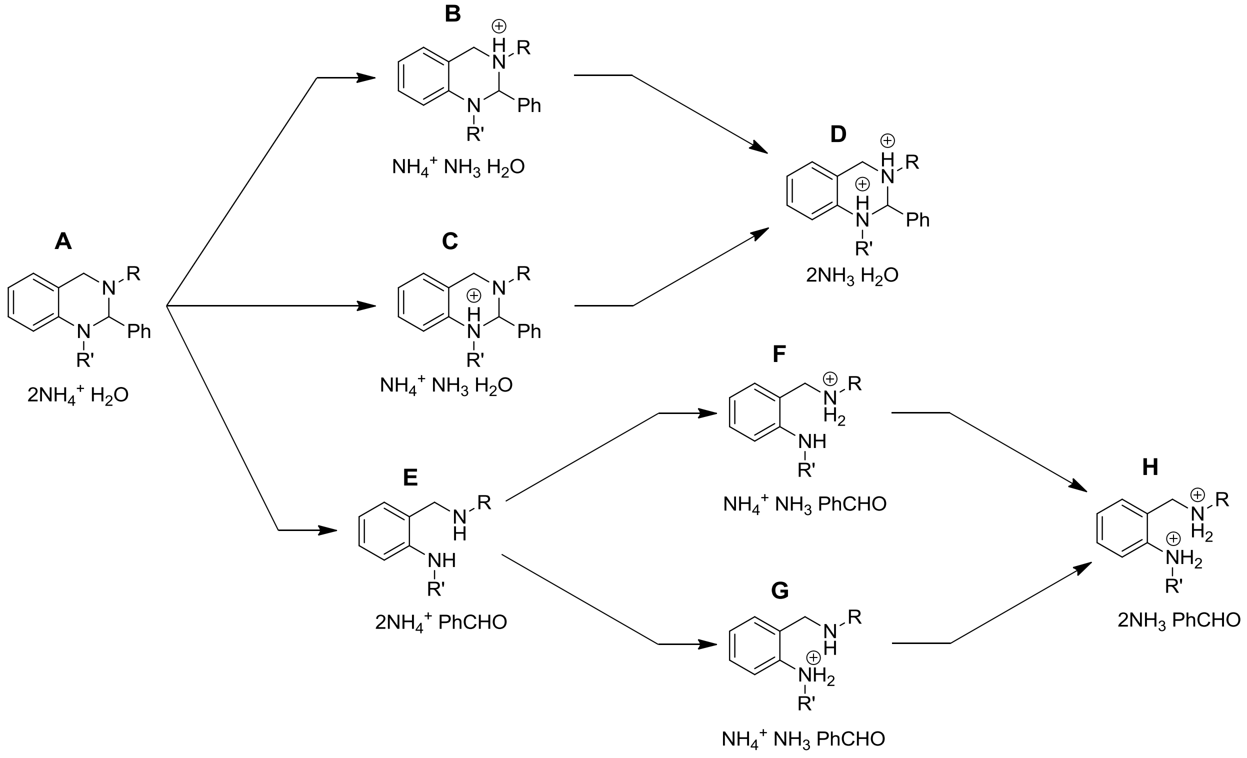

Table S2: Energy differences between states along the reaction pathway.

	Compound 8a:R = R′ = Me		Compound 13b: R = Me, R′ = iPr	
ΔU	B3LYP-D3 water (kcal/mol)	B3LYP-D3 gas (kcal/mol)	B3LYP-D3 water (kcal/mol)	B3LYP-D3 gas (kcal/mol)
B - A	4,68	-28,58	4,34	-30,24
C - A	13,47	-23,63	14,39	-25,36
D - B	31,36	71,33	33,19	66,46
D - C	22,56	66,37	23,15	61,58
E - A	3,11	2,78	-4,88	-6,72
F - E	-5,12	-35,89	-1,76	-32,64
G - E	-1,78	-34,75	2,08	-33,08
H - F	18,05	74,31	17,93	67,42
H - G	14,71	73,17	14,09	67,85
H - D	-20,00	-1,54	-26,25	-8,16
F - B	-6,69	-4,53	-10,99	-9,12
G - C	-12,14	-8,34	-17,20	-14,43

It is worth mentioning that in all calculations for both compounds, the N1 protonated open-ring diamine forms an intramolecular H bond, while the N3 protonated open-ring diamine of both compounds formed an intramolecular H bond only in the DFT optimisation in gas phase.

MM conformational search:

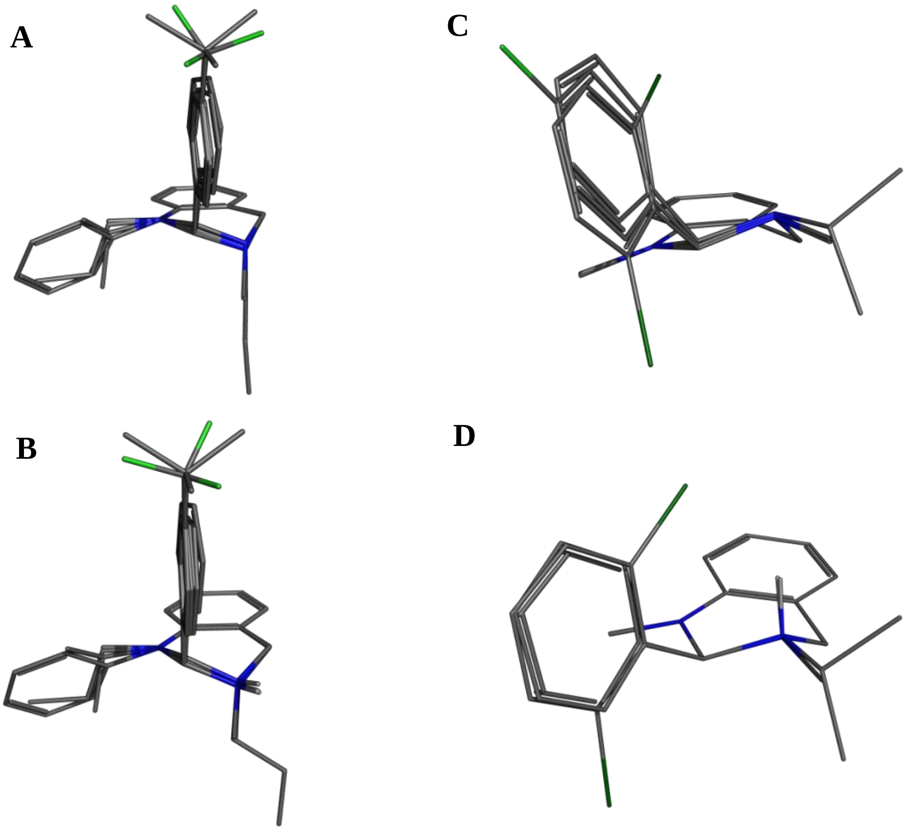

A: anti-axial conformation of minimal energy for neutral compounds (**8a–c**,**e**, **9a**, **13a–c**)
B: anti-axial conformation of minimal energy forN3 protonated compounds (**8a'-e'**, **9a'**, **13a'–c'**)
C: equatorial conformation of minimal energy for neutral compounds (**8d,f**, **9b**)
D: equatorial conformation of minimal energy for N3 protonated compounds (**8f'**, **9b'**)

Compounds	Number of Conformers	RMSD (Å)[a]	Motif of minimum energy conformation[b]
**8a**	2	0.0311	i (**A**)
**8c**	4	0.0207	i (**A**)
**8d**	2	0.0314	ii (**C**)
**8b**	4	0.0206	i (**A**)
**8e**	6	0.0187	i (**A**)
**8f**	5	0.0256	ii (**C**)
**9a**	74	0.0230	i (**A**)
**9b**	24	0.0331	ii (**C**)
**9c**	32	0.0208	i (**E**)
**13a**	69	0.0198	i (**A**)
**13b**	32	0.0283	i (**A**)
**13c**	27	0.0333	i (**A**)
**8a’**	4	0.0185	i (**B**)
**8c’**	9	0.0140	i (**B**)
**8d’**	4	0.0188	i (**B**)
**8b’**	9	0.0157	i (**B**)
**8e’**	9	0.0158	i (**B**)
**8f’**	3	0.0188	ii (**D**)
**9a’**	40	0.0115	i (**B**)
**9b’**	11	0.0178	ii (**D**)
**9c N1 prot**	22	0.0368	(**F**)
**13a’**	35	0.0156	i (**B**)
**13b’**	23	0.0216	i (**B**)
**13c’**	14	0.0298	i (**B**)
**8a N1 prot**	6	0.0258	(**G**)
**13b N1 prot**	20	0.0464	(**G**)
**8a diprot (anti)**	3	0.0494	(**H**)
**13b diprot (anti)**	13	0.0580	(**H**)

a: Mean value of the RMS for every conformer individually compared to the conformer of minimum energy after superposition.

b: i = anti-axial motif as described in the manuscript, ii = equatorial motif as described in the manuscript, () = letter corresponding to relative figure as presented in the Supporting Information.

Both the anti-axial and the equatorial conformers are found in all compounds, usually as next optimal conformation after the one of minimal energy. The energy difference between the respective other conformation is 2–5 kcal/mol.

In neutral compound **9c** the structure of minimal energy follows the anti-axial conformational motif, although N3 is more planar due to the presence of the phenyl group. The N1 protonated form of compound **9c** could not be superimposed with the rest of the compounds, as the protonation occurs in different atoms.

Conformers of minimal energy for **9c** neutral (**E**) and N1 protonated (**F**)

E

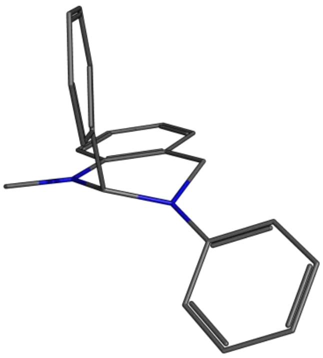

F

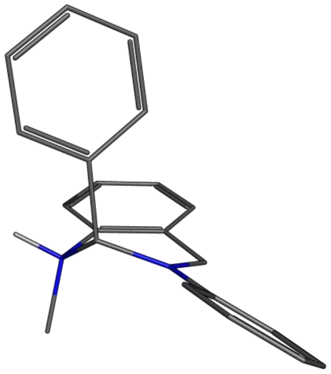

The systematic conformational search of the diprotonated compounds retained the protonated N stereoisomerism, therefore the syn- and anti- form of the N^+–H were both submitted for a conformational search and their minimal energy conformers were compared. The anti-conformer was of lower energy than the syn-, thus it was selected for the QM calculations.

Conformers of minimal energy for **8a** and **13b** N1 protonated (**G**) and anti-diprotonated (**H**)

G

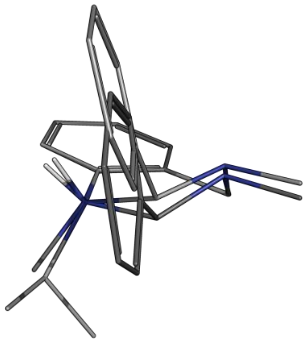

H

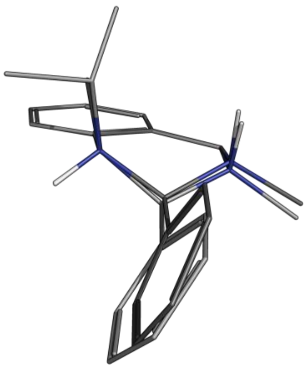

It is noteworthy to highlight that both the reference compound **8a** and the least stable compound **13b** share the same conformational motif in all cases - neutral from, N3 protonation, N1 protonation, and diprotonation.

MM ring strain comparison in gas phase

The energy values presented are calculated by: $\Delta U = U$ (**Cpd_mod** or **Cpd_mod'**) – U (**8a** or **8a'**).

ΔU [kcal/mol] gas					
	**8b_mod**	**8f_mod**	**9b_mod**	**13b_mod**	**13c_mod**
neutral	0.71	3.61	3.74	3.09	4.90
protonated	0.81	0.48	0.74	3.6	5.84

	U (kcal/mol) gas	*U* (kcal/mol) water
**8a**	-4.46	-11.09
**8a'**	-58.85	-106.01
**8b**	13.72	8.99
**8b_mod**	-3.75	-10.35
**8b'**	-41.02	-86.19
**8b'_mod**	-58.04	-105.28
**8f**	-16.45	-23.88
**8f_mod**	-0.85	-9.62
**8f'**	-84.20	-127.68
**8f'_mod**	-58.37	-104.40
**9b**	-1.65	-8.52
**9b_mod**	-0.72	-9.00

**9b’**	-54.64	-97.10
**9b’_mod**	-58.11	-103.55
**13b**	1.28	-4.04
**13b_mod**	-1.37	-8.04
**13b’**	-52.27	-97.41
**13b’_mod**	-55.21	-102.51
**13c**	38.41	33.76
**13c_mod**	0.44	-5.82
**13c’**	-13.02	-60.89
**13c’_mod**	-53.01	-100.79

Additional references

[1] Molecular Operating Environment (MOE), 2013.08; Chemical Computing Group Inc., 1010 Sherbooke St. West, Suite #910, Montreal, QC, Canada, H3A 2R7, 2015.

[2] Halgren, T. A. *J. Comput. Chem.* **1996**, *17*, 490–519.

[3] Goodman, J. M.; Still, W. C. *J. Comput. Chem.* **1991**, *12*, 1110–1117.

[4] Halgren, T. A. *J. Comput. Chem.* **1999**, *20*, 720–729.

[5] Becke, A. D. *J. Chem. Phys.* **1993**, *98*, 5648.

[6] Lee, C.; Yang, W.; Parr, R. G. *Phys. Rev. B* **1988**, *37*, 785–789.

[7] Vosko, S. H.; Wilk, L.; Nusair, M. *Can. J. Phys.* **1980**, *58*, 1200–1211.

[8] Stephens, P. J.; Devlin, F. J.; Chabalowski, C. F.; Frisch, M. J. *J. Phys. Chem.* **1994**, *98*, 11623–11627.

[9] Grimme, S.; Antony, J.; Ehrlich, S.; Krieg, H. *J. Chem. Phys.* **2010**, *132*, 154104.

[10] Eichkorn, K.; Weigend, F.; Treutler, O.; Ahlrichs, R. *Theor. Chem. Acc.* **1997**, *97*, 119–124.

[11] Häser, M.; Ahlrichs, R. *J. Comput. Chem.* **1989**, *10*, 104–111.

[12] Treutler, O.; Ahlrichs, R. *J. Chem. Phys.* **1995**, *102*, 346.

[13] Kendall, R. A.; Dunning, T. H.; Harrison, R. J. *J. Chem. Phys.* **1992**, *96*, 6796.

[14] Schäfer, A.; Huber, C.; Ahlrichs, R. *J. Chem. Phys.* **1994**, *100*, 5829.

[15] Klamt, A.; Schüürmann, G. *J. Chem. Soc., Perkin Trans. 2* **1993**, 799–805.

[16] Milletti, F.; Storchi, L.; Sforna, G.; Cruciani, G. *J. Chem. Inf. Model.* **2007**, *47*, 2172–2181.
